# Resistance to the isocitrate dehydrogenase 1 mutant inhibitor ivosidenib can be overcome by alternative dimer-interface binding inhibitors

**DOI:** 10.1038/s41467-022-32436-4

**Published:** 2022-08-15

**Authors:** Raphael Reinbold, Ingvild C. Hvinden, Patrick Rabe, Ryan A. Herold, Alina Finch, James Wood, Melissa Morgan, Maximillian Staudt, Ian J. Clifton, Fraser A. Armstrong, James S. O. McCullagh, Jo Redmond, Chiara Bardella, Martine I. Abboud, Christopher J. Schofield

**Affiliations:** 1 Chemistry Research Laboratory, Department of Chemistry and the Ineos Oxford Institute for Antimicrobial Research, University of Oxford, 12 Mansfield, Oxford, OX1 3TA UK; 2 Department of Chemistry, University of Oxford, Oxford, OX1 3QR UK; 3 Institute of Cancer and Genomic Sciences, University of Birmingham, Birmingham, UK; 4 Institute of Pharmaceutical Sciences, University of Freiburg, 79104 Freiburg, Germany; 5 GlaxoSmithKline, Gunnels Wood Rd, Stevenage, SG1 2NY UK; 6 Present Address: Institute of Genetics and Cancer, University of Edinburgh, Edinburgh, UK; 7 Present Address: Department of Natural Sciences, Lebanese American University, Byblos/Beirut, Lebanon

**Keywords:** X-ray crystallography, Acute myeloid leukaemia, Cancer therapeutic resistance, Oxidoreductases

## Abstract

Ivosidenib, an inhibitor of isocitrate dehydrogenase 1 (IDH1) R132C and R132H variants, is approved for the treatment of acute myeloid leukaemia (AML). Resistance to ivosidenib due to a second site mutation of IDH1 R132C, leading to IDH1 R132C/S280F, has emerged. We describe biochemical, crystallographic, and cellular studies on the IDH1 R132C/S280F and R132H/S280F variants that inform on the mechanism of second-site resistance, which involves both modulation of inhibitor binding at the IDH1 dimer-interface and alteration of kinetic properties, which enable more efficient 2-HG production relative to IDH1 R132C and IDH1 R132H. Importantly, the biochemical and cellular results demonstrate that it should be possible to overcome S280F mediated resistance in AML patients by using alternative inhibitors, including some presently in phase 2 clinical trials.

## Abstract (web summary)

The development of IDH variant inhibitors is a breakthrough as it is the first time metabolism has been successfully targeted by small molecule drugs in cancer. Here the authors report studies on resistance to the pioneer drug ivosidenib leading to identification of inhibitors retaining activity.

## Introduction

It has long been known that cancer cells have the potential for altered metabolism^1^, but it is only recently that this knowledge has been exploited for therapeutic benefit^1–3^. In humans, there are three isocitrate dehydrogenase isoforms (IDH1-3), which catalyse conversion of isocitrate to give 2-oxoglutarate (2-OG); IDH1/2 employ NADP^+^ as a cofactor, whereas IDH3, which is part of the tricarboxylic acid (TCA) cycle, uses NAD^+^^4,5^. Various somatic mutations to the genes encoding for isocitrate dehydrogenase 1 (IDH1) and 2 (IDH2) lead to variants with substantially increased capacity to catalyse reduction of 2-OG to 2-hydroxyglutarate (2-HG)^6–9^. Consequently, elevated 2-HG levels are proposed to promote tumorigenesis, potentially via chromatin destabilisation^10^. The most common IDH1 variants in cancer cells are R132H and R132C^11^.

Multiple IDH1 and IDH2 inhibitors are reported, though only one IDH1 inhibitor (ivosidenib) and one IDH2 inhibitor (enasidenib) have been approved for clinical use, i.e. for acute myeloid leukaemia (AML) treatment where the cancer cells make an IDH variant^12,13^. However, ivosidenib resistance has emerged as a consequence of a second-site mutation that produces IDH1 R132C/S280F, with 5 cases having been reported to date^14–16^.

The precise mechanistic basis by which the second site IDH1 S280F substitution enables resistance to ivosidenib and its consequences for IDH1 variant inhibition beyond that for ivosidenib have been unclear^14–16^. We report biochemical, structural, and cellular studies on IDH1 R132C/S280F and IDH1 R132H/S280F that address these questions. The results with isolated enzymes inform on the mechanism of S280F mediated resistance, which involves reduced inhibitor binding at the dimer-interface and alteration of kinetic properties, enabling enhanced 2-HG production relative to IDH1 R132C or R132H. Importantly, the results reveal that the S280F substitution causes resistance to ivosidenib, but this is not the case with several other IDH1 R132H and R132C inhibitors, some of which are in phase 2 clinical trials.

## Results

### The S280F substitution influences the biochemical properties of IDH1 R132C and R132H

**Fig. 1:**
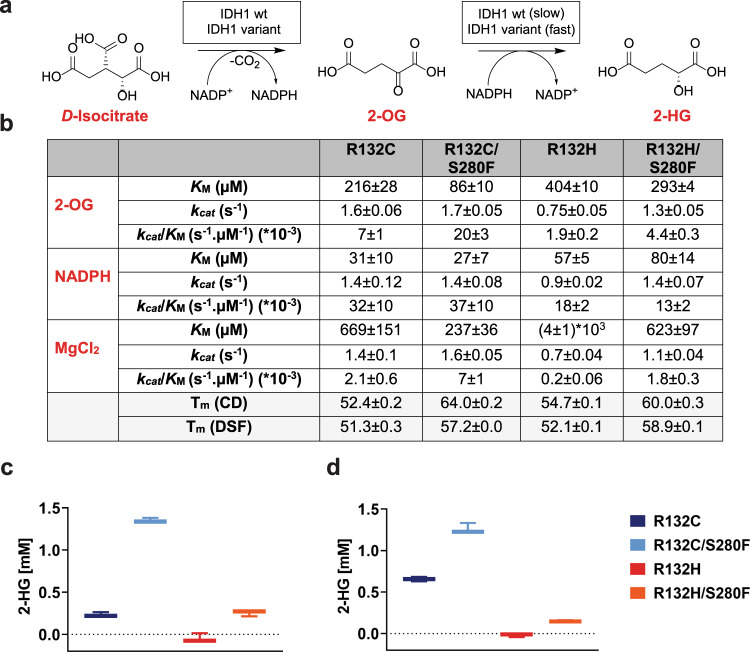
The IDH1 S280F substitution increases the efficiency of active site variants. **a** IDH1 variant catalysed oxidation of isocitrate and reduction of 2-OG to 2-HG. **b** Kinetic parameters for IDH1 variants (400 nM) from non-linear regression curve fits (standard error of the mean, *n* = 3). Conditions: 100 mM Tris, 10 mM MgCl_2_, 0.2 mM DTT, 0.005%_v/v_ Tween 20, and 0.1 mg/mL BSA (pH 8.0). *Shaded:* Melting temperatures (T_m_s) of IDH1 variants measured by differential scanning fluorimetry (DSF, 3 µM enzyme) or circular dichroism (CD, 0.2 mg/mL enzyme); λ: 215 nm. Conditions: DSF (20 mM Tris, 100 mM NaCl, pH 7.4); CD (10 mM sodium phosphate, pH 8.0). See Supplementary Information for details. **c** 2-HG formation from 2-OG catalysed by IDH1 variants as measured by ^1^H NMR (700 MHz; error bars: standard errors of the mean, *n* = 3 independent replicates of ^1^H time course experiments). Conditions: 500 nM enzyme, 10 mM MgCl_2_, 1.5 mM 2-OG, 1.5 mM NADPH; incubation time: 12 min. Source data are provided as a Source Data file. **d** 2-HG formation from isocitrate catalysed by IDH1 variants as measured by NMR (700 MHz; standard error of the mean, *n* = 3 independent replicates of ^1^H time course experiments). Conditions: 750 nM enzyme, 10 mM MgCl_2_, 3 mM DL-isocitrate, 1.5 mM NADP^+^; incubation time: 9 min. Buffer: 50 mM Tris-d_11_, 100 mM NaCl, 10 mM MgCl_2_, and 10% D_2_O, pH 7.5. Source data are provided as a Source Data file.

To investigate the mechanism of IDH1 S280F mediated inhibitor resistance, we used established protocols^17,18^ to produce highly purified forms of recombinant IDH1 R132C/S280F and R132H/S280F and, for comparison, IDH1 R132C, IDH1 R132H, IDH1 wildtype (wt), IDH1 S280F (without IDH1 R132C or R132H) and IDH1 R132H/Q277E (Supplementary Fig. 1a). The IDH1 R132H/Q277E variant was made because acquired drug resistance against the IDH2 mutant inhibitor enasidenib has been linked to (i) IDH2 R140Q/I319M and (ii) IDH2 R140Q/Q316E; IDH2 I319 is homologous to IDH1 S280 and IDH2 Q316 is homologous to IDH1 Q277 (Supplementary Fig. 1b/c)^14^. Consistent with prior studies on IDH1 R132H and R132C, all the recombinant proteins were predominantly dimeric in solution (Supplementary Fig. 1d–f) and likely copurify with two NADPH molecules (as reported for IDH1 wt and R132H^18^, and shown for IDH1 R132C, Supplementary Fig. 1g/h). Whilst biophysical studies show that the S280F substitution does not affect the secondary structure (Supplementary Fig. 1i), this substitution substantially enhances the thermodynamic stability of the IDH1 R132C and R132H variants, as well as IDH1 wt (Fig. 1b; Supplementary Fig. 1j). Note that in the subsequent text, all IDH variants referred to are IDH1-based, except where stated otherwise.

We analysed the catalytic activities of the R132C/S280F and R132H/S280F variants, employing ^1^H NMR to measure the reduction of 2-OG to 2-HG and the two-stage turnover of isocitrate to 2-HG (Fig. 1a/c/d; Supplementary Fig. 2a/b). Due to the overall redox-neutral nature of the two reactions involved in converting isocitrate to 2-HG, this conversion cannot be readily monitored by NADP^+^/NADPH measurements^18^. As anticipated, the NMR results show *D*-isocitrate to be a substrate for R132C/S280F and R132H/S280F (Supplementary Fig. 2c). Notably, they reveal that both R132C/S280F and R132H/S280F catalyse conversion of isocitrate to 2-HG and of 2-OG to 2-HG more efficiently than R132C or R132H, respectively. (Fig. 1c/d)

Kinetic analyses with R132C/S280F and R132H/S280F monitoring NADPH consumption by UV spectroscopy (Fig. 1b) demonstrated that the S280F substitution increases the catalytic efficiency (as measured by *k*_*cat*_/*K*_M_) for the 2-OG to 2-HG conversion, compared to the analogous R132C and R132H variants, with decreased *K*_M_ values for both 2-OG and Mg^2+^. By contrast with these variants, the R132H/Q277E variant, which is predominantly dimeric, and which has a similar secondary structure compared to the other IDH variants studied (Supplementary Fig. 1d–f, i), was inactive by ^1^H NMR assays (Supplementary Fig. 2d/e) and is less stable than R132H (Supplementary Fig. 1j; Fig. 1b). Differential scanning fluorimetry (DSF) studies using N-oxalylglycine (NOG) as a catalytically inactive 2-OG analogue^19,20^, and isocitrate (with 10 mM Mg^2+^), indicate that R132H/Q277E does not, at least efficiently, bind NOG or isocitrate, likely reflecting its inactivity (Supplementary Fig. 2f).

Kinetic analyses measuring NADP^+^ consumption for the S280F variant (without the R132C or R132H substitutions) for conversion of isocitrate to 2-OG (Supplementary Table 1a) show increased efficiency (*k*_*cat*_/*K*_M_) compared to IDH1 wt as a result of a decreased *K*_M_ for isocitrate; there was no change in the apparent *K*_M_ for Mg^2+^. However, with the IDH1 S280F variant, the *K*_M_ values for 2-OG and Mg^2+^ (100-fold) were both decreased for conversion of 2-OG to 2-HG (Supplementary Table 1b), while the *k*_*cat*_ was decreased 10-fold compared to IDH1 wt. The kinetic results for the conversions of isocitrate and 2-OG measured through NADP^+^ or NADPH turnover are broadly consistent with the ^1^H NMR turnover assays (Supplementary Fig. 3a–f). The results showing that the S280F substitution decreases the *K*_M_ values for both substrates and Mg^2+^ (apart from the conversion of isocitrate to 2-OG as catalysed by IDH1 S280F) are notable because recent work has shown that allosteric dimer-interface binding of IDH1 variant inhibitors hinders both Mg^2+^ and substrate binding^18^.

To further investigate the mechanistic consequences of the S280F substitution, we studied the IDH1 variants using cyclic voltammetry (Supplementary Fig. 3g–l), which can measure rates in each NADP(H) cycling direction and which can distinguish the conversion of isocitrate to 2-OG from the reduction of 2-OG to 2-HG^17^. The results imply that R132C and R132C/S280F catalyse the conversion of isocitrate to 2-OG with similar efficiency (Supplementary Fig. 3g/h). However, the efficiency of the overall conversion of isocitrate to 2-HG is enhanced for R132C/S280F compared to R132C (Fig. 1d), with the conversion rate of 2-OG to 2-HG being approximately doubled compared to IDH1 R132C (Supplementary Fig. 3g/h). The cyclic voltammetry results show that compared to R132H, R132H/S280F displays increased efficiency (approximately twofold), both for the conversion of isocitrate to 2-OG and of 2-OG to 2-HG (Supplementary Fig. 3i/j). IDH1 S280F (without R132C or R132H) displays a twofold increased reaction rate compared to IDH1 wt for conversion of isocitrate to 2-OG (Supplementary Fig. 3k/l).

Overall, although the three turnover assays measuring NADPH absorbance, ^1^H NMR resonances, or cyclic voltammetry parameters employed different conditions, they all show that the S280F substitution improves the efficiency of the R132C and R132H variants in converting isocitrate to 2-HG, principally by increasing the rate of reduction of 2-OG to 2-HG.

Previous studies have shown that most IDH variant inhibitors, likely including ivosidenib (there is no reported crystal structure for ivosidenib complexed to an IDH1 variant), bind at the IDH1/IDH2 dimer-interface^21–26^. We have recently shown that inhibitor-mediated disruption of Mg^2+^ binding occurs in a manner that disproportionally affects the conversion of 2-OG to 2-HG, compared to the wildtype reaction of isocitrate to 2-OG^18^. Consistent with prior results using NOG and isocitrate, DSF analyses imply that Mg^2+^ is required for efficient binding of isocitrate and 2-OG to R132C, R132C/S280F, R132H, and R132H/S280F (Supplementary Fig. 3m/n). It is possible that the S280F substitution increases the affinity of Mg^2+^ binding, at least for R132C/R132H catalysed conversion of 2-OG to 2-HG.

### Ivosidenib does not inhibit isolated IDH1 double variants, but several other inhibitors retain activity

**Fig. 2:**
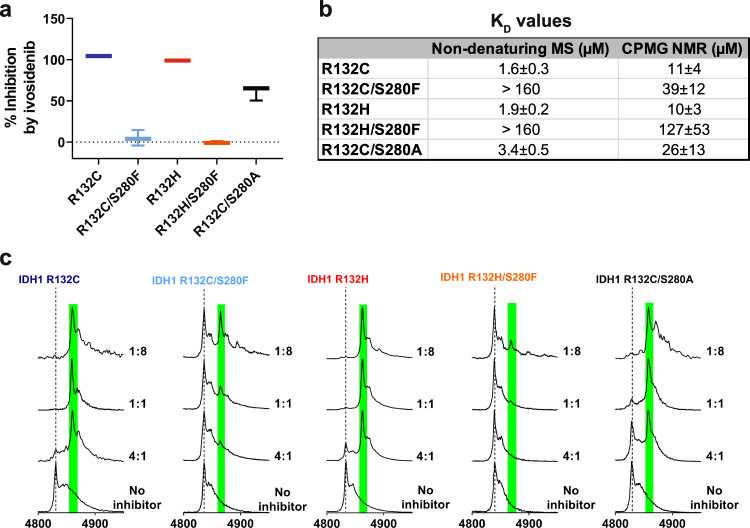
The S280F substitution reduces ivosidenib inhibition and binding. **a** Inhibition (%) of IDH1 variants (400 nM) by ivosidenib (10 µM) as determined by the NADPH absorbance assay. Errors: standard errors of the mean (*n* = 3 independent replicates measured on the same 96-well plate). Source data are provided as a Source Data file. **b** K_D_s determined by non-denaturing MS (20 µM enzyme, technical errors: *n* = 3 for the *z* = 19, 20, 21 charge states) and using the CPMG Project pulse NMR sequence (10 µM enzyme, *n* = 2). Non-denaturing MS: ammonium citrate buffer (200 mM, pH 7.5); CPMG NMR: 50 mM Tris-d_11_, 100 mM NaCl, 10 mM MgCl2, and 10 % D_2_O, pH 7.5. **c** Non-denaturing MS analysis measuring binding of ivosidenib to IDH1 variants. At 20 µM IDH1 variants are predominantly dimeric with 2 NADP(H) molecules bound - dashed line. Green background corresponds to binding of one ivosidenib molecule. Final concentrations of ivosidenib: 5 µM (4:1), 20 µM (1:1), and 160 µM (1:8). Cone-voltage: 100 V.

We then investigated inhibition of isolated R132C, R132C/S280F, R132H, R132H/S280F by ivosidenib (Fig. 2a; Supplementary Fig. 4a). NMR and MS analyses both demonstrate that the S280F substitution reduces the binding efficiency of ivosidenib (Fig. 2b) and that the stoichiometry of ivosidenib binding is one inhibitor molecule per dimer (Fig. 2c); these observations are consistent with prior work on IDH1 wt and R132H inhibition by ivosidenib^18^. It has been proposed, based on modelling studies^15,16^, that resistance to ivosidenib is mediated by hindrance to inhibitor binding due to substitution of S280 with a more sterically demanding phenylalanine (S280F), and that ivosidenib binding is additionally hindered by the loss of a hydrogen bond to the alcohol side chain of S280^16^. To investigate whether resistance is solely mediated by steric hindrance or other factors are involved, including those related to a potential loss of a hydrogen bond to S280, we produced R132C/S280A. Ivosidenib does not (at least, efficiently) inhibit R132C/S280F or R132H/S280F (Fig. 2a), but inhibits R132C/S280A with an IC_50_ 992 nM, compared to IC_50_ 2.5 nM for R132C (Supplementary Fig. 4b). This observation suggests that steric hindrance by the phenylalanine side chain and the loss of a hydrogen bond to S280 may both contribute to ivosidenib resistance (R132C/S280A is less potently inhibited than R132C while there is no inhibition of R132C/S280F) (Fig. 2a; Supplementary Fig. 4b). This proposal is consistent with inhibitor binding studies employing non-denaturing MS and CPMG NMR (Fig. 2b), which show a decrease in the affinity of R132C/S280A for ivosidenib compared to R132C, but that the decrease is more substantial for R132C/S280F (Fig. 2b).

The combined results show that the S280F substitution both weakens binding of ivosidenib and promotes conversion of 2-OG to 2-HG. Notably, the second site S280F substitution also promotes, at least when compared with the single R132C or R132H substitutions, conversion of isocitrate to 2-HG in the absence of inhibitor (Fig. 1d). Interestingly, IDH1 S280F (without R132C or R132H) has increased catalytic efficiency for turnover of isocitrate to 2-OG, but not for the reduction of 2-OG to 2-HG compared to IDH1 wt (Supplementary Table 1a/b; Supplementary Fig. 3k/l), suggesting potential co-operativity between the two substitution sites with respect to increasing the efficacy of the overall conversion of isocitrate to 2-HG. For both isocitrate and 2-OG, substrate binding (as judged by *K*_M_) is increased in IDH1 S280F (Supplementary Table 1a/b). For the reduction of 2-OG to 2-HG, the *K*_M_ of Mg^2+^ for IDH1 S280F is decreased 100-fold compared to IDH1 wt, whereas it does not change for conversion of isocitrate to 2-OG. These combined observations imply that the S280F substitution promotes substrate (isocitrate or 2-OG) binding, but the effects on Mg^2+^ binding depend on the reaction catalysed, i.e. isocitrate to 2-OG, or 2-OG to 2-HG. This conclusion is consistent with recent work showing that binding of substrates and Mg^2+^ to IDH1 variants involves conformational changes, including movements of the α-helices (α9 and α10) that are involved in forming the dimer-interface^18^; note there is evidence that IDH1 manifests half-site reactivity^27^. Notably, ivosidenib and, at least, some other IDH variant inhibitors can also bind to IDH1 wt (and likely IDH2 wt), but do not efficiently inhibit them^18^. These observations illustrate the complexity of the conformational dynamics at the IDH1 dimer-interface and how they are linked to the active site chemistry. These subtle mechanistic issues, including potential modulation of Mg^2+^ and substrate binding, make it difficult to predict precisely how allosteric ligand binding at the dimer-interface site will manifest in terms of inhibition. We thus screened a set of 14 reported IDH variant inhibitors against R132C/S280F and R132H/S280F with isolated enzymes (Supplementary Fig. 4a/c).

**Fig. 3:**
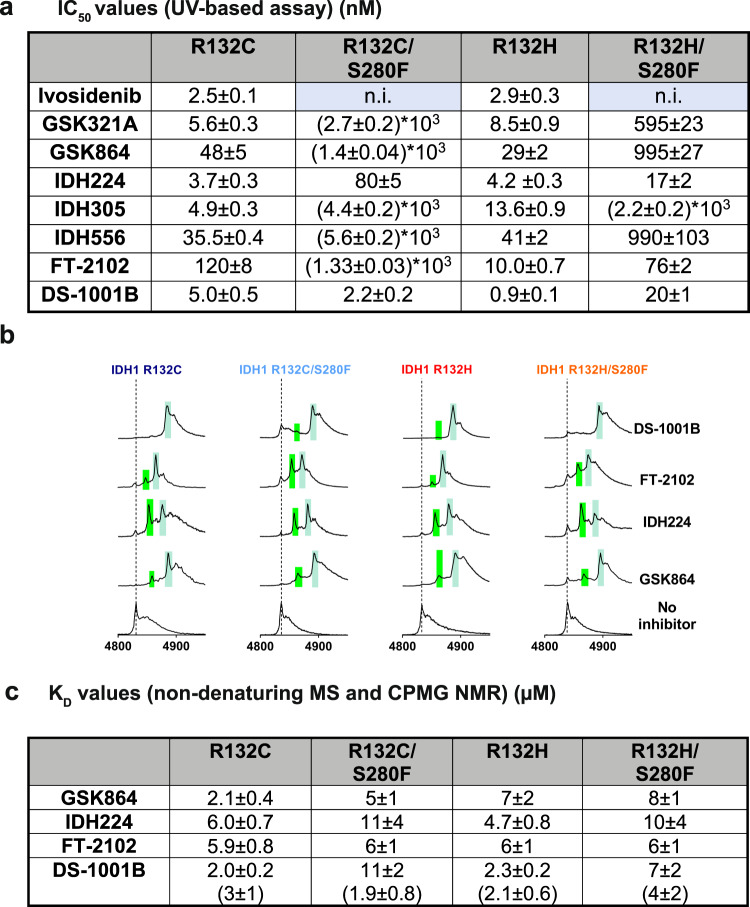
The S280F substitution causes changes in inhibitor potency varying with the active site substitution and type of inhibitor. **a** IC_50_ values (standard error of the mean, *n* = 3) with IDH1 variants (at 30 nM); n.i. = no inhibition observed. Conditions: 100 mM Tris, 10 mM MgCl_2_, 0.2 mM DTT, 0.005%_v/v_ Tween 20, and 0.1 mg/mL BSA (pH 8.0). **b** Non-denaturing MS analyses. Dashed line: IDH1 dimer (*z* = 20, with 2 NADP(H) molecules bound), green shading corresponds to binding of one inhibitor molecule, mint shading corresponds to binding of 2 inhibitor molecules. Conditions: 20 µM IDH1 variant, 20 µM inhibitor, cone-voltage: 100 V. **c** K_D_ determinations (standard errors of the mean) measured by by non-denaturing MS (20 µM enzyme, technical errors: *n* = 3 for the *z* = 19, 20, 21 charge states) and CPMG NMR (values in brackets, 10 µM enzyme, *n* = 2). Non-denaturing MS: ammonium citrate buffer (200 mM, pH 7.5); CPMG NMR: 50 mM Tris-d_11_, 100 mM NaCl, 10 mM MgCl_2_, and 10% D_2_O, pH 7.5.

Importantly, although seven of the inhibitors did not manifest inhibition of R132C/S280F or R132H/S280F (including one, SYC-435, reported to bind at the active site^28^), the screening results (Supplementary Fig. 4c) revealed that seven of the inhibitors retained activity against the double variants, i.e. GSK321, GSK864, IDH224, IDH305, IDH556, FT-2102, and DS-1001B. Some of these inhibitors manifest high potency (IC_50_s < 100 nM), i.e. IDH224, FT-2102 (only against R132H/S280F), and DS-1001B (Fig. 3a). Potent representative inhibitors from each inhibitor series were selected for further studies (i.e. GSK864, IDH224, FT-2102, DS-1001B). Strikingly, non-denaturing MS studies showed that all these inhibitors can bind with a stoichiometry of 2 molecules to each IDH1 dimer (Fig. 3b; Supplementary Fig. 4d). Note that this observation contrasts with that for ivosidenib, where only one inhibitor molecule was observed to bind to the IDH1 dimer (Fig. 2c). The inhibitor binding affinity (determined by non-denaturing MS) was generally lower for R132C/S280F and R132H/S280F than R132C and R132H, respectively although it is not altered for FT-2102 (Fig. 3c). The most potent R132C/S280F inhibitor, DS-1001B, was also analysed in a solution assay (employing CPMG NMR) and was found to manifest tight binding to all the tested variants, with the lowest affinity for R132H/S280F (K_D_ = 4.19 µM) compared to the other tested IDH1 variants (Fig. 3c).

Turnover assays employing the NADPH absorbance assay imply that R132C, R132C/S280F, R132H, and R132H/S280F inhibition is apparently competitive with respect to Mg^2+^ and 2-OG levels; inhibitor potency was not substantially influenced by variations in NADPH concentrations, except potentially with FT-2102 (Supplementary Fig. 5a–c).

### Crystallographic analyses suggest underlying mechanisms for properties of IDH1 double variants and their inhibition

**Fig. 4:**
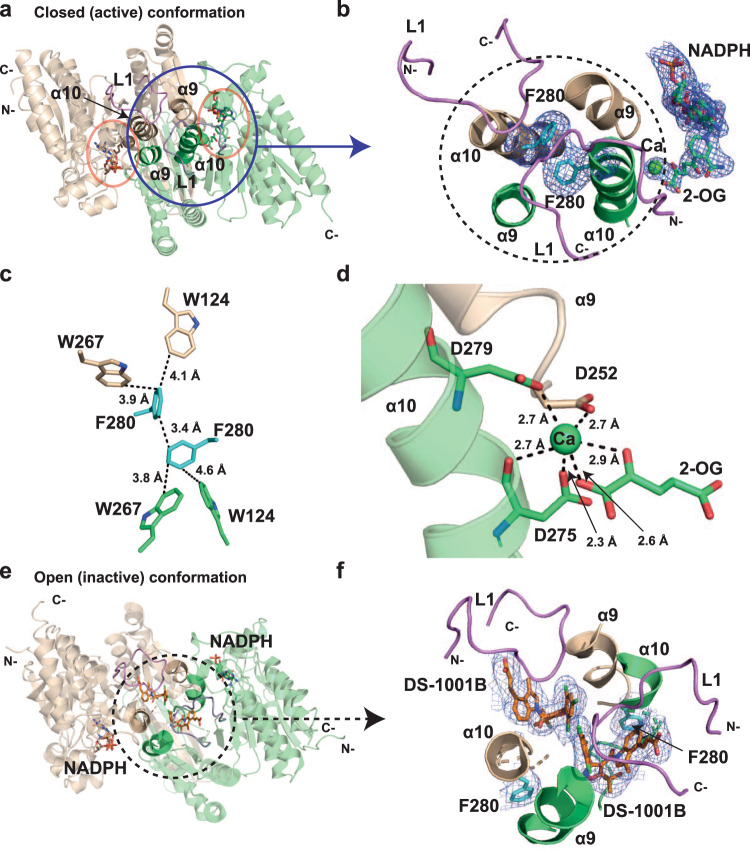
Crystallographic analyses reveal that the S280F substitution affects the dimer-interface and active site of IDH1 variants. **a** Ribbon view from a crystal structure of R132C/S280F (PDB: 7PJM, 2.1 Å resolution). As observed in the asymmetric unit, an apparent dimer is formed by chain A (wheat) and chain B (green). Orange circles: active sites with 2-OG, NADPH, and calcium. L1 loop: violet. **b** Close-up view with a Polder omit map (blue mesh, contour 3.0 σ) showing 2-OG, NADPH, and calcium. The L1 loop covering the dimer-interface is in violet. Black circle: dimer-interface with a Polder omit map (blue mesh, contour: 3.0 σ) showing F280 (cyan). **c** View of the dimer-interface highlighting hydrophobic interactions between W124 (L1), F280 (cyan, α10), and W267 in both monomers. **d** View of the metal-binding site. The calcium-binding residues D252 (monomer 1, wheat, α9), D275 and D279 (monomer 2, green, α10) are shown. **e** Ribbon view from a crystal structure of R132C/S280F (PDB: 7PJN), 2.45 Å resolution) complexed with 2 NADPH and 2 DS-1001B (orange) molecules; the enzyme is likely in an open inactive conformation. An apparent dimer is formed by chain B (wheat) and chain C (green), as observed in the asymmetric unit. DS-1001B binds at the dimer-interface (black circle). **f** Polder omit map (blue mesh, contour: 3.0 σ) showing DS-1001B and residue F280 (cyan) in the dimer-interface. Note that the L1 loop (violet) covers the dimer-interface.

To further investigate the effects of the S280F substitution on inhibitor binding, we worked to acquire crystallographic information and obtained a structure of R132C/S280F complexed with calcium, 2-OG and NADPH (2.1 Å resolution, space group: C222_1_, three molecules (A–C) in the asymmetric unit (Supplementary Fig. 6a), PDB: 7PJM; the structure was solved using a reported structure of R132H, as a search model^19^; Fig. 4a–d).

Comparison with reported IDH1 structures^19,29^ implies that the R132C/S280F structure likely reflects a closed active site conformation (the widths of the two active site clefts in the apparent homodimer formed by chains A and B measured by the distances between I76 and L250, which are residues at the entrance of the active site^29^, are 12.6 Å and 13.0 Å, indicating a closed conformation^29^; Supplementary Fig. 6b/c). The structure reveals that the phenyl rings of the two S280F residues, located on α10, are adjacent to each other at the dimer-interface (Fig. 4a/b); together with the side chains of W124 and W267, the phenyl group of S280F creates an increased hydrophobic region at the dimer-interface (Fig. 4c) possibly reflecting the increased thermal stabilities of the S280F variants (Fig. 1b; Supplementary Fig. 1j).

In the R132C/S280F structure, it is notable that the L1 loop, which partially covers the inhibitor binding pocket^30^ (Fig. 4b; Supplementary Fig. 7a/b), adopts a different conformation compared to that observed in an R132H crystal structure complexed with calcium, 2-OG and NADPH^19^. Although the difference in the L1 loop conformation might (in part) reflect different crystallisation conditions, this observation supports the importance of conformational motions during IDH1 catalysis and inhibition. Further, there are differences in the conformation of the metal-binding residue Asp-252 (Fig. 4d; Supplementary Fig. 7c/d) in the R132C/S280F structure compared to those observed in other structures, including R132H^19^. However, the overall nature of 2-OG binding, which includes chelation by the calcium ion, is unchanged in the different structures (Supplementary Fig. 7d).

We obtained a crystal structure of R132C/S280F complexed with NADPH and its potent inhibitor DS-1001B (2.45 Å resolution, space group: P2_1_2_1_2, four molecules (A–D) in the asymmetric unit (Supplementary Fig. 8a), PDB: 7PJN; the structure was solved using a reported inhibitor-bound structure of R132H as a search model^31^; Fig. 4e/f). Consistent with results from non-denaturing MS (Fig. 3b), the structure reveals two DS-1001B molecules bound at the dimer-interface (Fig. 4e). Interestingly, the two DS-1001B molecules bind in a manner such that their trichlorophenyl rings are adjacent, an observation of interest with respect to future medicinal chemistry efforts aimed at improving inhibitor binding (Fig. 4f). The DS-1001B complex structure likely reflects an open or semi-open (inactive) conformation: the widths of the two active site clefts in the apparent homodimer formed by chain B and chain C as measured by distances between I76 and L250, are 17.7 Å (semi-open) and 20.2 Å (open; Supplementary Fig. 8b/c). As previously reported for the inactive conformation^23^, α10 in both monomers is partly disordered and assumes a partial loop conformation (Supplementary Fig. 9), which potentially helps to accommodate inhibitor binding. Notably, by contrast with the structure of R132C/S280F without inhibitor, in the DS-1001B complexed structure, the S280F residues of the two monomers are not adjacent but 14.9 Å apart (closest C-atom, Supplementary Fig. 10).

Although care should be taken not to overinterpret differences in crystal structures obtained under different conditions, consistent with the solution studies, our crystallographic results show, that the S280F substitution both affects the chemistry at the dimer-interface where ivosidenib likely binds and that it has potential to affect the active site chemistry including with respect to metal ion binding, and as a consequence, substrate binding. When combined with other structures and recent kinetic and other biophysical studies^18,27^, these biophysical results further highlight the role of conformational dynamics and complexity in IDH (variant) catalysis. Defining the precise nature of these and their inhibition effects is challenging and requires structural studies on individual turnovers coupled with modelling. Hence, we propose that IDH variant inhibition, including with respect to combatting resistances, should be principally pursued by an empirically guided approach employing different types of turnover and binding assays linked to cellular studies at an early stage.

### Cellular studies on IDH1 R132C/S280F and R132H/S280F demonstrate potent inhibition in a cellular environment

**Fig. 5:**
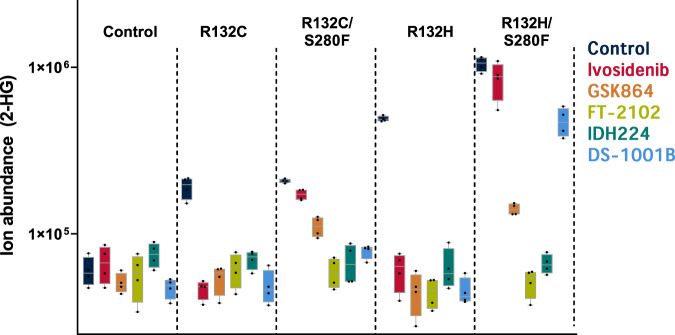
Influence of inhibitors on 2-HG levels in cells bearing recombinant R132C, R132C/S280F, R132H and R132H/S280F. LN18 cells were treated with the inhibitors ivosidenib, GSK864, FT-2102, IDH224, and DS-1001B in DMSO (final concentration: 5 µM). 2-HG levels were determined by anion-exchange chromatography coupled to MS^51^ (errors: standard errors of the mean, *n* = 4 independent replicates). Note that, whilst ivosidenib is not/poorly active in reducing 2-HG levels in R132C/S280F and R132H/S280F bearing cells, the other inhibitors are active in reducing 2-HG levels. Control cells were generated by transduction with lentiviral vectors containing no IDH1. Box-and-whisker plots: The centre line is the median and the bounds are 25th and 75th percentile values. The whiskers are the minimum and maximum measured 2-HG levels for each experimental group. Source data are provided as a Source Data file.

Support for this approach comes from the results of cellular studies on five selected (potential) R132C/S280F inhibitors, i.e. ivosidenib, GSK864, IDH224, FT-2102, and DS-1001B (Fig. 5). An LN-18 glioblastoma cell line (ATTC CRL-2610) was used to produce recombinant IDH1 variants (R132C, R132C/S280F, R132H, R132H/S280F) as reported in Supplementary Fig. 11.

As anticipated, with control cells, which do not express any IDH1 variants, the inhibitors had no detectable effect, within error, on the low levels of the endogenous 2-HG present (Fig. 5). Consistent with the reported results^24,26,32^, all the inhibitors efficiently suppressed production of 2-HG in cells over-producing R132C and R132H. By contrast, the effects of the five inhibitors on cells over-producing R132C/S280F or R132H/S280F varied. Consistent with the biochemical data showing that the S280F substitution causes resistance to ivosidenib and prior clinical results^14–16^, ivosidenib was ineffective in suppressing 2-HG production in the R132C/S280F and R132H/S280F overproducing cells (Fig. 5). In the R132C/S280F overproducing cells, GSK864 was only moderately potent in reducing 2-HG levels, whereas IDH224, DS-1001B, and FT-2102 were more potent in lowering 2-HG levels. Interestingly, FT-2102 was relatively less potent than IDH224 and DS-1001B against isolated R132C/S280F (Fig. 3a; IC_50_ 1.3 µM). The relatively high potency of FT-2102 in cells may reflect enhanced cell permeability compared to IDH224 and DS-1001B.

In the case of R132H/S280F (Fig. 5), the results for ivosidenib, GSK864, IDH224, and FT-2102 treatment were analogous to those for R132C/S280F bearing cells. Strikingly, however, DS-1001B was a relatively poor inhibitor of R132H/S280F in this cellular context. This difference likely reflects, at least in part, the higher IC_50_ for DS-1001B versus isolated R132H/S280F compared to values for the other tested IDH1 variants (Fig. 3a).

Although there are differences in the relative efficiencies of the inhibitors, as described above, the cellular results correlate well with the results for isolated IDH1 variants. Most importantly, they reveal that specific inhibitors, including FT-2102 and DS-1001B, which are in clinical phase 2 trials^33–35^, are effective in reducing 2-HG levels in R132C/S280F bearing cells.

## Discussion

The development of IDH variant inhibitors is a breakthrough in cancer treatment as it is a pioneering example of how metabolism can be successfully targeted by small-molecule drugs. However, as anticipated, resistance has emerged to the pioneer drug ivosidenib, in particular, via the S280F substitution at the dimer-interface^14–16^. Inhibitor screening results with isolated IDH1 variants reveal that neither the R132C/S280F, nor R132H/S280F variants, are (efficiently) inhibited by ivosidenib. Importantly, however, this result is also the case for some, but not all, of the reported IDH variant inhibitors currently in development. Thus, it should be possible to overcome IDH variant inhibitor resistance caused by dimer-interface mutants by appropriate medicinal chemistry programmes.

The combined biochemical and structural results show that the S280F substitution enables resistance to ivosidenib in cancer cells producing R132C/S280F or R132H/S280F in part by hindering its binding to the IDH1 dimer-interface and in part by increasing the efficiency of R132C and R132H variants in catalysing conversion of isocitrate and/or 2-OG to 2-HG. The studies reported here and elsewhere^18^ imply that allosteric binding of IDH variant inhibitors at the dimer-interface affects active site Mg^2+^ (or Mg^2+^-substrate complex in the case of isocitrate) binding in a manner that disproportionally inhibits 2-OG to 2-HG conversion compared to isocitrate to 2-OG conversion. The precise molecular reasons for these observations, along with the details of IDH1 wt catalysis, should be the subject of future time-resolved biophysical studies.

Although the in vivo cancer relevance of the observed increased catalytic efficiency of R132C S280F in vitro is unknown, it may help to maintain elevated 2-HG levels in the presence of an IDH variant inhibitor. If so, it supports a functional role for 2-HG in mature cancer cells and consequently inhibitor based treatment resulting in a reduction in 2-HG levels; note that the role of 2-HG in mature cancer cells may be different or in addition to the proposed role of elevated 2-HG levels in tumorigenesis^36,37^.

Despite the mechanistic complexity of the IDH1 variant inhibition by allosteric binding, our results clearly show that R132C/S280F mediated resistance to ivosidenib can be overcome by using other inhibitors, e.g. IDH224, FT-2102, DS-1001B, and likely improved variants of these. Like ivosidenib, these inhibitors also bind at the dimer-interface; however, unlike ivosidenib, which binds with a stoichiometry of one inhibitor molecule per IDH1 variant dimer, the potent R132C/S280F inhibitors (as shown by biochemical and cellular studies) bind with a stoichiometry of two inhibitor molecules per dimer as shown by non-denaturing MS studies and crystallographic analyses of R132C/S280F with and without an inhibitor. Importantly, two inhibitors of R132C/S280F and R132H/S280F, FT-2102 and DS-1001B are already in phase 2 clinical trials^33–35^. There is thus the possibility of developing efficient treatment regimens involving the substitution of one IDH variant inhibitor for another as resistance emerges. There is also the possibility of using combinations of inhibitors, in particular when the types of resistance that will likely arise can be predicted prior to initial treatment.

Recent studies on the mechanisms of IDH1 and its variants and the mode of action of reported IDH variant inhibitors^18^ have led to the suggestion that allosteric inhibitors were preferentially identified in part as a consequence of the screening conditions used, i.e. the screens were not carried out with varied Mg^2+^ concentrations. The nature of S280F enabled ivosidenib resistance to R132C/S280F suggests that the development of new types of IDH inhibitors, including ones that do not work by allosteric binding to the dimer-interface, is of interest. It is possible that the binding of inhibitors at the IDH dimer-interface, which is involved in subtle conformational changes during catalysis, might be particularly prone to resistance compared to those binding at the active site and/or competing with NADPH. It should, however, be noted that the S280F substitution confers resistance to an inhibitor, SYC-435, which is reported to bind at the IDH1 active site^28^.

Due to the limited available clinical data at this stage, it is difficult to predict how resistance will emerge to IDH variant inhibitors. We thus suggest that along with optimisation of the currently effective inhibitors, which bind at the dimer-interface, the development of IDH variant inhibitors working via mechanisms not involving allosteric binding is worthwhile. Our results also imply that future medicinal chemistry efforts should optimally employ multiple IDH1/2 variants, including resistance-enabling mutations in isolated form (under different assay conditions) as well as empirically guided cell and in vivo analyses.

## Methods

### Site-directed mutagenesis

Forward and reverse primers to introduce different mutations were designed according to reported procedures^38^. A pET22b plasmid (Sigma-Aldrich) was used as a template. IDH1 R132H/S280F and R132C were made from an IDH1 R132H DNA template (obtained as described^18^). IDH1 R132C/S280F and R132C/S280A were made from an R132C DNA template. IDH1 S280F was made from an IDH1 wt DNA template. Reactions were conducted (2-step method) using the Q5^®^ High-Fidelity DNA Polymerase (New England Biolabs), deoxynucleotide solution mix (New England Biolabs) and a GeneAmp PCR System 9700 (Applied Biosystems). The mixture was purified using the GeneJET PCR Purification Kit (Thermo Scientific) according to the manufacturer’s protocol. The template DNA was digested using Dpn1 (New England Biolabs; 3 h incubation, 37 °C). The product was transformed into XL10-gold cells (Agilent). Colonies were grown overnight in 10 mL 2TY medium, and the plasmid was extracted using the GeneJET Plasmid Miniprep Kit (Thermo Scientific). The plasmids were eluted with MilliQ purified water. Sequences were confirmed by Sanger sequencing conducted by Eurofins Scientific.

### Protein production

Recombinant protein production was conducted as described^17,18^. In brief, the recombinant proteins were produced in BL21(DE3)plysS *E.coli* cells with 1 mM Isopropyl β- d-1-thiogalactopyranoside (IPTG) with a post-induction temperature of 20 °C. The cells were harvested by centrifugation and lysed by sonication. The cell lysates were loaded onto a 5 mL HisTrap HP column (Cytiva) and eluted with an imidazole step gradient (up to 500 mM). Fractions containing the desired protein were further purified using a Superdex S200 column (GE Healthcare, 300 mL) by isocratic elution. The purity of fractions was assessed using SDS PAGE gel electrophoresis (Supplementary Fig. 1a). Protein concentrations were determined using a Nanodrop One machine (Thermo Scientific) and correspond to the IDH1 monomer concentration.

For crystallographic studies, IDH1 R132C/S280F was additionally purified by anion-exchange chromatography. After initial purification using a 5 mL HisTrap HP column (Cytiva), fractions containing the desired protein were subjected to buffer exchange using a PD-10 column (Merck) and loaded onto a Cytiva Q Sepharose Fast Flow column. The protein was eluted with a NaCl gradient (up to 1 M) then subjected to gel filtration purification (Superdex S200 column (GE Healthcare, 300 mL), with isocratic elution using buffer containing 20 mM Tris and 100 mM NaCl (pH 7.4).

For electrochemical experiments, Ferredoxin-NADP^+^ reductase (FNR) from *Chlamydomonas reinhardtii* was produced as described^39^. In brief, recombinant FNR was produced in BL21(DE3)plysS *E. coli* cells by adding 1 mM IPTG, and the cultures were grown for a further 4 h (37 °C). The cells were harvested by centrifugation; the pellets were resuspended in a small volume of cold buffer (50 mM HEPES, 150 mM NaCl, 1 mM DTT; pH 7.4) and stored overnight at −80 °C. The cells were thawed and lysed using a french press at 20 psi, and the lysate was centrifuged at 4 °C using an ultracentrifuge (Beckman L8-70M Ultracentrifuge). FNR was isolated from the supernatant using a Ni^2+^ HisTrap HP affinity column (GE Healthcare Life Sciences) and eluted from the column using an imidazole gradient (final concentration of 250 mM imidazole). Fractions containing FNR were selected based on the absorbance at 280 nm and 460 nm. The fractions containing FNR were combined and concentrated using Amicon Ultra-4 mL Centrifugal filters (10 kDa) to a final volume of around 2 mL. The concentrated FNR solution was then applied to a desalting column (PD-10; GE Healthcare) to remove the imidazole. The enzyme was separated into single-use aliquots, flash-frozen in liquid nitrogen, then stored at −80 °C.

### Spectrophotometric assays

Enzyme kinetics were determined spectrophotometrically based on changes in the concentration of NADPH as measured by its absorption at 340 nm (ε = 6220 M^–1^ cm^–1^). Analyses were conducted in 96-well half area clear microtiter plates (Greiner Bio-One 675001) using a PHERAstar FS Microplate Reader at 25 °C in a reaction volume of 100 µL. This assay was used to study steady-state kinetics and inhibition of reactions catalysed by IDH1 variants. The assay buffer was 100 mM Tris, 10 mM MgCl_2_, 0.2 mM dithiothreitol (DTT), 0.005%_(v/v)_ Tween 20, and 0.1 mg mL^−1^ bovine serum albumin (BSA) (pH 8.0). For inhibition assays, IDH1 variants were incubated with an inhibitor for 12 min before the reaction was initiated by substrate addition. The standard error was derived from curve fitting using GraphPad Prism v9. See figure legends for details on specific kinetic assays (Figs. 1–3; Supplementary Fig. 5; Supplementary Table 1).

2-Oxoglutarate (2-OG) disodium salt, NADPH tetrasodium salt (~98%), NADP^+^ disodium salt, *D*-isocitrate potassium salt, and MgCl_2_ × 6 H_2_O were from Sigma-Aldrich. *DL*-isocitric acid trisodium salt was from ChemCruz. Inhibitors were from MedChemExpress, BioVision, DC Chemicals, Sigma-Aldrich, Enzo Life Sciences, Cambridge Bioscience Ltd. GSK321 and GSK864 were kindly provided by GSK. Stock solutions were prepared in DMSO (10 mM).

### ^1^H NMR studies

IDH1 variants were buffer exchanged into NMR buffer (50 mM Tris-d_11_, 100 mM NaCl, 10% D_2_O, pH 7.5) using Micro Bio-Spin 6 columns (Bio-Rad) according to the manufacturer’s protocol. Nuclear Magnetic Resonance (NMR) spectra were obtained using a Bruker AVIII 700 MHz NMR spectrometer equipped with an inverse 5 mm TCI ^1^H/^13^C/^15^N cryoprobe. MgCl_2_ (10 mM), isocitrate/2-OG and NADP^+^/NADPH were added for turnover assays, and the turnover was monitored using ^1^H NMR (NS: 16, Relaxation delay: 2 s). The water signal was suppressed by excitation sculpting. The data were analysed using Mestrenova.

### Carr-Purcell-Meiboom-Gill (CPMG) NMR

IDH1 variants were buffer exchanged into NMR buffer (50 mM Tris-d_11_, 100 mM NaCl, 10% D_2_O, pH 7.5) and concentrated using Amicon Ultra Centrifugal Filters (0.5 mL, cut off: 50 kDa) according to the manufacturer’s protocol. Experiments were conducted with 10 µM inhibitor (from a 10 mM d_6_-DMSO stock). The IDH1 variant was titrated into this solution, and the PROJECT-CPMG sequence was applied^40^. Experimental parameters were as follows: total echo time, 40 ms; relaxation delay, 2 s. Water suppression was achieved by pre-saturation. The percentage of protein-inhibitor complex ([PL]/([P] + [PL]) was plotted against inhibitor concentration. The data were analysed using Mestrenova and the dissociation constant was calculated using non-linear regression by GraphPad Prism (version 9).

### Circular dichroism studies

IDH1 variants were buffer exchanged into sodium phosphate buffer (10 mM, pH 8.0) using Micro Bio-Spin 6 Columns (Bio-Rad) according to the manufacturer’s protocol. CD measurements used a Chirascan CD spectrometer (Applied Photophysics) equipped with a Peltier temperature-controlled cell holder. The spectra were recorded in a range from 260 to 185 nm (0.5 nm intervals). Measurements were conducted in triplicates at 23 °C, and the background was subtracted from that of the sample. The spectra were averaged and smoothed using the Savitzky–Golay filter (Window size 4). Data were normalised to the protein concentration (measured using a NanoDrop One machine), and the mean residue ellipticity was calculated according to the formula [Eq. 1]:

1 $$\begin{array}{c}{{{{{\rm{MRE}}}}}}=\frac{{{{{{\boldsymbol{\theta }}}}}}}{{{{{{\bf{10}}}}}}\ast {{{{{\boldsymbol{l}}}}}}\ast {{{{{\boldsymbol{N}}}}}}\ast {{{{{\boldsymbol{C}}}}}}}\,[{{{\rm {deg}} }}.{{{{{{\rm{cm}}}}}}}^{2}.{{{{{{\rm{dmol}}}}}}}^{-1}] \\ {{\theta }}{:}\,{{{{{\rm{Degree}}}}}}\,{{{{{\rm{of}}}}}}\,{{{{{\rm{ellipticity}}}}}}\\ {{{{{\rm{N}}}}}}{:}\,{{{{{\rm{Number}}}}}}\,{{{{{\rm{of}}}}}}\,{{{{{\rm{amino}}}}}}\,{{{{{\rm{acids}}}}}}\\ {{{{{\rm{C}}}}}}{:}\,{{{{{\rm{Concentration}}}}}}\,({{{{{\rm{mol}}}}}}.{{{{{{\rm{L}}}}}}}^{-1})\\ {{{{{\rm{l}}}}}}{:}\,{{{{{\rm{Path}}}}}}\,{{{{{\rm{length}}}}}}\,(0.1\,{{{{{\rm{cm}}}}}}).\end{array}$$

To analyse the thermal stability of the IDH1 variants, the wavelength was fixed at 215 nm, and the temperature gradually increased from 10 to 80 °C. The heating rate was 1 °C min^−1^, and the CD signal was measured every 2 °C (±0.4 °C). Spectra were smoothed using the Savitzky–Golay filter (4th order polynomial, 12 neighbours) of GraphPad Prism (version 9). The melting temperature was calculated using a Boltzmann sigmoidal model with Graph Pad Prism (version 9); the standard error was derived from curve fitting using the Boltzmann sigmoidal model.

### Differential scanning fluorimetry (DSF)

Measurements were conducted in Tris buffer (50 mM, pH 7.4) with 3 µM protein and 3× Sypro Orange on a Bio-Rad Thermal Cycler CFX96. The heat rate was +0.2 °C s^−1^ in a range of 20–95 °C. Data were analysed using Bio-Rad CFX Manager 3.1. The standard error was derived from three independent replicates.

### Non-denaturing gel analysis

Novex^™^ WedgeWell^™^ 4–12% Tris-glycine gels were used. The gel chamber (Invitrogen) was cooled in an ice bath, and the gel electrophoresis was conducted in a cold room (4 °C) using NativePAGE running buffer (Invitrogen). The gel electrophoresis was carried out for 1.5 h at 160 V, and the gel was stained using InstantBlue Coomassie (Expedeon).

### Size exclusion chromatography—Multi-angle laser scattering (SEC-MALS)

SEC-MALS measurements were conducted using a Wyatt Dawn HELEOS-II 8-angle light scattering detector and Wyatt Optilab rEX refractive index monitor linked to a Shimadzu HPLC system comprising LC-20AD pump, SIL-20A Autosampler and SPD20A UV/Vis detector. Samples were analysed with a Superdex 200 HR10/30 column with a flow rate of 0.5 ml min^−1^. The sample concentration was 1 mg mL^−1^ in a buffer containing Tris (20 mM, pH 7.4) and NaCl (100 mM).

### Electrochemical experiments

Electrochemical experiments were performed in an anaerobic glovebox (Glove Box Technology) containing a nitrogen atmosphere (O_2_ < 1 ppm). The electrochemical apparatus (a glass electrochemical cell and rotating pyrolytic graphite edge (PGE) electrodes) were as previously reported^17^. An Autolab PGSTAT 10 potentiostat using Nova software was used to conduct electrochemical experiments. Electrode potentials (*E*) were measured against a saturated calomel electrode (SCE) and converted to the standard hydrogen electrode (SHE) using a temperature-dependent potential conversion equation (at 25 °C the conversion is: *E*_SHE_ = *E*_SCE_ + 0.241 V)^17^. The counter electrode was a platinum wire. Nanoporous indium tin oxide (ITO) electrodes were made by electrophoretically depositing ITO nanoparticles (<50 nm, Sigma-Aldrich) onto pyrolytic graphite edge electrodes (ITO/PGE)^17^. Enzymes were loaded onto the electrode by drop-casting a 4–6 µL mixed enzyme solution onto the ITO electrode and allowing it to incubate at room temperature for at least 30 min while ensuring that the solution did not evaporate. In all cases, 0.85 nmol (homodimer basis) of IDH1 (wildtype and all variants) was used; the amount of FNR that was co-loaded was adjusted to achieve the desired final enzyme ratio. All tested neomorphic IDH1 variants (R132C, R132C/S280F, R132H, R132H/S280F) were loaded at a 2.5 to 1 molar ratio with FNR (i.e. 2.5-fold more (homodimer molar equivalent) of each IDH1 variant was loaded relative to FNR for each experiment). By contrast, IDH1 wt and IDH1 S280F were loaded at a 1 to 8 molar ratio with FNR to ensure the system was not FNR-limited due to these IDH1 enzymes oxidising *DL*-isocitrate at a much faster rate than the neomorphic IDH1 variants. IDH1 concentrations were calculated based on their homodimers. Electrodes loaded with enzyme were thoroughly rinsed using buffer solution before submerging them in the reaction buffer for each experiment to ensure there was no free enzyme in solution.

### Non-denaturing mass spectrometry

IDH1 variants were buffer exchanged into non-denaturing MS buffer (ammonium acetate, 200 mM, pH 7.5) using Micro Bio-Spin 6 Columns (Bio-Rad) according to the manufacturer’s protocol. Non-denaturing MS experiments were carried out using a quadrupole-TOF (Waters Synapt G2Si) instrument and an Advion Triversa Nanomate chip-based ESI autosampler. Inhibitors were dissolved in MeOH and added to the protein solution (final protein concentration: 20 µM). A spray voltage of 1.7–1.8 kV (spray backing gas pressure 0.6 psi, inlet pressure 3.7 mbar) was applied. The sample cone voltages were 100 V and 5.2 V. Spectra were analysed using Mass Lynx (version 4.1), including centring and smoothing. The percentage of protein-inhibitor complex ([PL]/([P] + [PL]) was plotted against inhibitor concentration. A baseline correction was applied, and the dissociation constant was calculated using non-linear regression by GraphPad Prism (version 9).

### Crystallisation and X-ray structure determinations

#### Closed active conformation

IDH1 R132C/S280F (25.62 mg mL^−1^ in 20 mM Tris, 100 mM NaCl, pH 7.4) was used for crystallisation using the sitting drop vapor diffusion method. For the sitting drop setup, a 24 well Cryschem Plate (Hampton Research, USA) with a reservoir solution of 250 µL was used^19^. A screen varying the PEG concentration (PEG3350 15–20%, horizontal axis in steps of 1%) and the salt concentration (calcium acetate 200 or 225 mM, vertical axis; seeded or unseeded, respectively) and Bis-Tris (0.1 M) at pH 7 was carried out. The enzyme (25 µL) was incubated for 1 h on ice with 10 mM NADPH (in H_2_O, 10 µL), 20 mM CaCl_2_ (in H_2_O, 5 µL) and 200 mM 2-OG (in H_2_O, 10 µL) to yield a final protein concentration of 12.81 mg mL^−1^. Crystallisation was achieved by the addition of 2 µL of the protein containing solution to a 2 µL precipitant solution. The plates were sealed with StarSeal Advanced Polyolefin Film (Starlab, Germany); crystals (100 µM average size) were manifest within 14 days. To obtain crystals reproducibly, seeding was employed with crushing of crystals using the SeadBeat kit (Hampton Research, USA) according to the manufacturer’s protocol. Crystal containing droplets were cryo-protected by mixing them in a ratio of 1:1 with reservoir solution containing glycerol (25%_(v/v)_), harvested with a nylon loop, and cryo-cooled in liquid N_2_. The crystals were stored in liquid N_2_ until required for data collection.

Data were collected at 100 K using the Diamond Light Source (DLS) beamline I24. Data were indexed, integrated, and scaled using the Xia2^41^ strategy of the beamline auto-processing pipeline (Supplementary Table 4). The IDH1 R132C/S280F crystal structure was determined by molecular replacement (MR) using the AutoMR (PHASER^42^) subroutine in PHENIX^43^. The search model used for MR was based on IDH1 R132H (PDB: 4KZO^19^). The structural model was optimised by iterative cycles of manual rebuilding in COOT^44^ and crystallographic refinement in Phenix.refine^45^ (refinement details are summarised in Supplementary Table 4).

### Open inactive conformation with inhibitor DS-1001B

IDH1 R132C/S280F (25.62 mg mL^−1^ in 20 mM Tris, 100 mM NaCl, 1 mM tris(2-carboxyethyl)phosphine (TCEP), pH 7.4) was used for crystallisation. For the sitting drop setup, a 24 well Cryschem Plate (Hampton Research, USA) with a reservoir solution of 250 µL was used as reported^25^. A screen varying the ammonium citrate concentration (pH 7.0, 0.75–2 M, horizontal axis in steps of 0.25 M) and the DTT concentration (1.5–3 mM, vertical axis in steps of 0.5 mM) was carried out. The protein (12.5 µL) was incubated on ice for 1 h with NADPH (10 mM; in buffer containing 20 mM Tris, 100 mM NaCl, 1 mM TCEP, pH 7.4; 5 µL), buffer (6.8 µL), and a 10-fold excess of DS-1001B (in DMSO, 0.7 µL) to a final protein concentration of 12.81 mg mL^−1^. 2 µL of this solution was then added to 2 µL precipitant solution. The sitting drop plate was sealed with StarSeal Advanced Polyolefin Film (Starlab, Germany), and the crystals appeared after one day. Crystal harvesting was performed with glycerol as a cryoprotectant as described above.

Data were collected at 100 K using synchrotron radiation at DLS beamline I03. Data were indexed, integrated, and scaled using the Xia2^41^ strategy of the beamline auto-processing pipeline (Supplementary Table 4). An initial MR solution was obtained using an inhibitor-bound structure of IDH1 R132H (PDB: 5TQH^31^). A starting model was re‐built from this MR solution using PHENIX AutoBuild^45–49^. The structural model was optimised by iterative cycles of manual rebuilding in COOT^44^ and refinement using Phenix.refine^45^ (details are summarised in Supplementary Table 4). Due to the limited resolution (2.45 Å), NCS restraints were used throughout and TLS refinement of B-factors (16 TLS groups for the four chains).

### Lentiviral expression of IDH1 variants in LN18 cell lines

#### Molecular cloning and cell transduction with lentiviral vectors

Human glioblastoma LN18 cells were obtained from the ATCC and cultured according to the supplier’s instructions. LN18 cells were genetically modified to overexpress transgenes encoding for IDH1 R132H, IDH1 R132C, IDH1 R132H/S280F or IDH1 R132C/S280F using lentiviral vector transduction. First, the IDH1 S280F mutant sequence of IDH1 was generated by a recombinant PCR-based approach, using the lentiviral transfer vectors pCC.sin.36.IDH1R132H.PPTWpre.CMV.tTA-S2tet and the pCC.sin.36.IDH1R132C.PPTWpre.CMV.tTA-S2tet^50^, containing the R132H and R132C sequences of IDH1, as templates. In this reaction, Primer1_forward and Primer1_reverse (Supplementary Table 2) were used. The IDH1 S280F mutant amplicon was subsequently subcloned into the same transfer vectors using BamH1-HF and Nhe1-HF. Subsequently, the IDH1 R132H, R132C, IDH1 R132H/S280F or IDH1 R132C/S280F sequences were amplified in PCR reactions using Primer2_forward and Primer2_reverse (Supplementary Table 2) and cloned into to the pUltra-Chili vector (AddGene), using Xma1 and NheI-HF. Restriction enzymes were from New England Biolabs. All bacterial transformations were performed using XL10-Gold ultracompetent cells according to the manufacturer’s instructions. All constructs were verified by Sanger sequencing using Primer3_forward and Primer3_reverse (Supplementary Table 2).

### Generation of lentiviral vectors and cell transduction

All the IDH1 mutant pUltra-Chili transfer plasmids were packaged into lentiviral vectors (LVs) by transient transfection of HEK293T cells along with the 3 packaging plasmids: pVSVg, pREV and pMDL. HEK293T cells were cultured in IMDM (Sigma, I3390) supplemented with 10% FBS (Fisher Scientific, 11550356) and 5% pen-strep antibiotic (Sigma, P4458). 48 h after transfection, HEK293T conditioned medium was harvested, centrifuged and filtered using 0.22 µm filters. The viral p24 antigen concentration was measured using an HIV-1 p24 core profile enzyme-linked immunosorbent assay ELISA assay Lenti-X p24 Rapid Titer kit according to the manufacturer’s instructions. Serial dilutions of freshly harvested conditioned medium were used to infect 1.2 × 10^5^ LN18 cells in a six-well plate in the presence of polybrene (8 μg ml^−1^). As a control, cells were transduced with pUltra-Chili lentiviral vector, containing TdTomato, but no IDH1 sequences.

### RNA extraction and RT-PCR, and qPCR

RNA was extracted using the RNeasy Micro Kit (Qiagen) according to the manufacturer’s instructions. When required, complementary DNA was reverse transcribed using the High-Capacity cDNA Reverse Transcription Kit (Applied Biosystems). qPCR was used to measure IDH1 expression in control and LV-transduced cells using an IDH1 TaqMan probe (Life Technologies) and the TaqMan Fast Universal PCR Master-Mix (Applied Biosystems). The reaction was performed using the QuantStudio^™^ 5 Dx Real-Time PCR System (Applied Biosystems) with GAPDH serving as an endogenous control (Life Technologies). Each target gene’s expression was evaluated using a relative quantification approach (2 − ΔΔCT method).

### Tissue culture and inhibitor treatment using genetically modified LN18 cell lines

Liquid and sterile filtered Dulbecco’s phosphate-buffered saline (PBS), foetal bovine serum (FBS) of non-US origin, and Dulbecco’s Modified Eagle’s Medium (DMEM) with 4500 mg L^−1^ glucose and sodium bicarbonate, without *L*-glutamine, were from Merck Life Sciences. The GlutaMAX^TM^ supplement was from Thermo Fisher Scientific. Dimethyl sulfoxide (DMSO) for molecular biology was from Merck. Sterile syringe filters (15 mm diameter, 0.2 µM pore, RC membrane) were from Corning. Inhibitors were dissolved in DMSO to 5 mM concentration and filtered before use with Corning^®^ syringe filters (regenerated cellulose, 18 mm diameter, 0.2 µm pores).

LN18 cells containing an empty vector or producing recombinant IDH1 R132C, IDH1 R132C/S280F, IDH1 R132H, or IDH1 R132H/S280F were incubated at 37 °C and 5% CO_2_ and grown in DMEM (4500 mg L^−1^ glucose) supplemented with 10% (v/v) FBS and 1% (v/v) GlutaMAX^TM^. Cells were seeded into 12-well plates, with 0.7 mL per well at 200,000 cells mL^−1^. After 24 h of incubation, the original medium was replaced with fresh 0.7 mL medium (DMEM (4500 mg L^−1^ glucose), 10% FBS and 1% GlutaMAX^TM^) with either 5 µM inhibitor (ivosidenib, GSK864, IDH224, FT-2102, DS-1001B) or 0.1% DMSO (control samples). Cells were incubated for a further 24 h prior to harvesting. The medium was removed by aspiration during harvest before the wells were gently washed twice with 0.7 mL PBS per well. 75 µL of 80%_(v/v)_ aqueous methanol was added to each well, and the plate was placed on dry ice. The cells were scraped, and the content of each well was transferred to separate microtubes.

### Sample preparation and MS studies

Cell extracts were centrifuged at 14,000 *g* for 25 min. The DNA concentration (ng µL^−1^) of the supernatants were measured using a ClarioStar Plus with an LVis plate (260 nm). 2.5 µL of the supernatant was added to each well. The plate was blanked with 2.5 µL 80%_(v/v)_ aqueous methanol per well prior to measurement of DNA concentration. The remaining cell sample supernatant was transferred to Total Recovery vials (Waters) and diluted relative to DNA concentration. Anion-exchange chromatography-MS analysis was carried out as reported^51^ using a Dionex ICS-5000+ high-pressure ion chromatography system equipped with a continuously regenerated trap column, Dionex ERS 500e suppressor and AS11-HC (2 × 250 mm, 4 μm) column, all from Dionex (Sunnyvale, CA, USA). This was coupled directly to a Q-Exactive HF hybrid quadrupole-Orbitrap MS via a HESI II probe, both from Thermo Fisher. The column temperature was 30 °C, and all samples were injected with a 5 µL partial loop injection. The mobile phase was aqueous hydroxide ions (flow rate: 0.250 mL min^−1^) with the following gradient: 0 min, 0 mM; 1 min, 0 mM; 15 min, 60 mM; 25 min, 100 mM; 30.1 min, 0 mM; 37 min, 0 mM. The suppressor had a flow rate of 0.500 mL min^−1^ and was used in the external water mode. The MS was operated in negative ion mode with the following source parameters: sheath gas flow rate, 60; auxiliary gas flow rate, 20; sweep gas flow rate, 0; spray voltage 3.6 kV; capillary temperature, 300 °C; S-lens RF level, 70; heater temperature, 350 °C. MS and MS/MS scan parameters were: microscans, 2; resolution, 7 × 10^4^; AGC target, 1 × 10^6^ ions; maximum IT, 250 ms; loop count, 10; MSX count, 1; isolation window, 2.0 m/z; collision energy, 35; minimum AGC target, 5 × 10^3^ ions; apex trigger 1–15 s; charge exclusion 3–8, >8; dynamic exclusion, 20.0 s. The retention time of 2-HG was determined by analysis of 1 µg mL^−1^ standard in 80% _(v/v)_ aqueous methanol (HPLC grade, from Sigma-Aldrich).

## Supplementary information

Supplementary Info File #1

## Data Availability

The crystallographic data generated in this study have been deposited in the PDB database under accession codes 7PJM and 7PJN. Source data are provided with this paper. Any additional information required to reanalyse the data reported in this paper is available from the corresponding authors upon request. Source data are provided with this paper.

## Source data

Source Data
