# Supplementary material for: Resistance to the isocitrate dehydrogenase 1 mutant inhibitor ivosidenib can be overcome by alternative dimer-interface binding inhibitors
Source: Nat Commun. 2022 Aug 15;13:4785. doi: 10.1038/s41467-022-32436-4 (PMC9378673; doi:10.1038/s41467-022-32436-4)

Supplementary Information

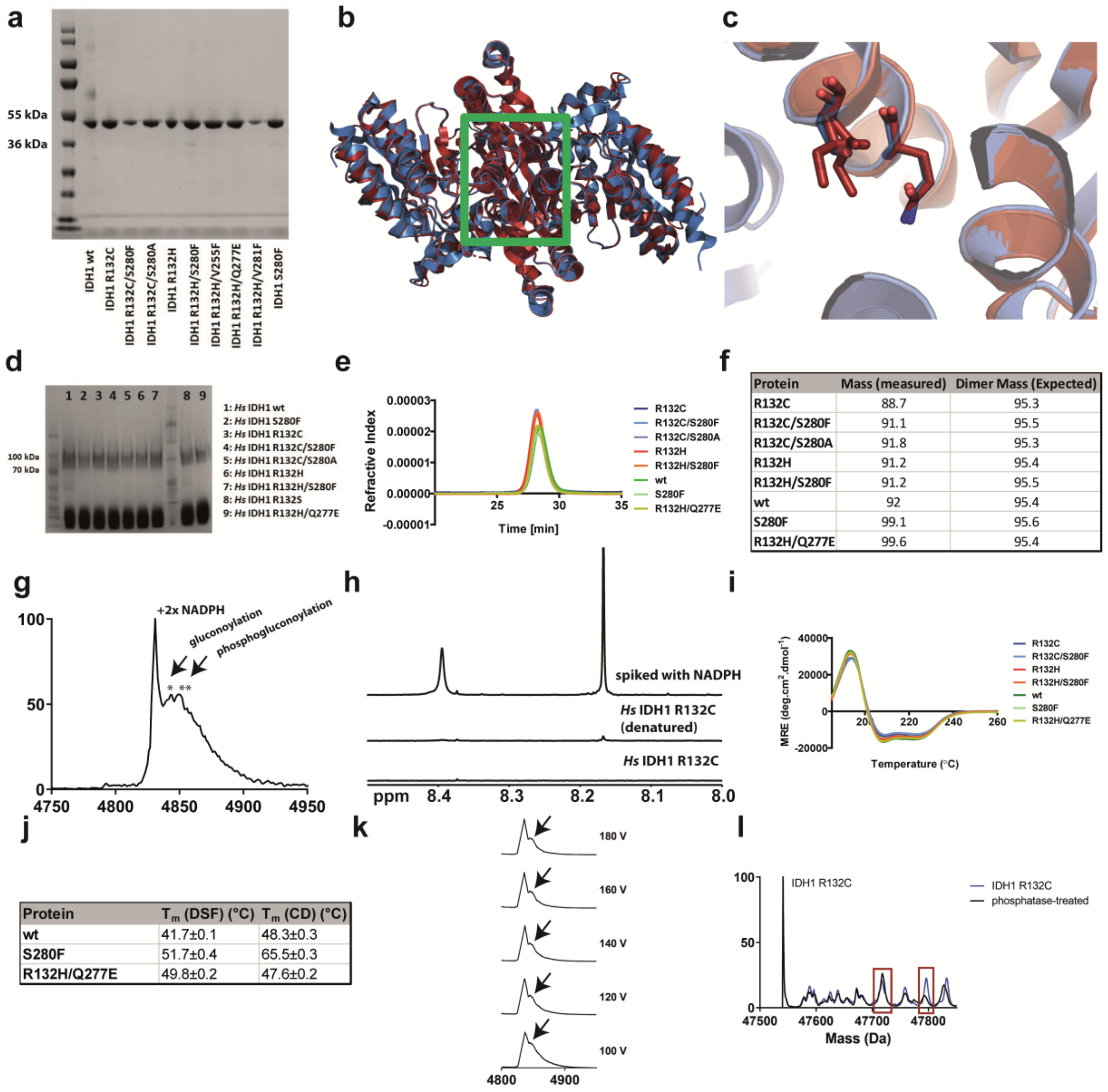

Supplementary Figure 1 | Analyses of recombinantly produced IDH1 variants. ***(a)*** *SDS-PAGE analyses of purified recombinant IDH1 variants. Note the IDH1 R132H/V255F and R132H/V281F variants were not the subjects of this study. This analysis was repeated independently once with similar results.* ***(b)*** *Superimposition of IDH1 R132H (blue, PDB ID: 4KZO[1]) and IDH2 R140Q (red, PDB ID: 5I95[2]) structures. The dimer-interface is highlighted in green.* ***(c)*** *Note that α-helices α9 and α10 are at the centre of the dimer-interface. IDH1 S280 is analogous to IDH2 I319. IDH1 Q277 is analogous to IDH2 Q316.* ***(d)*** *Non-denaturing gel analyses of IDH1 variants using a non-denaturing Tris-glycine gel. All variants display a single band between 70 kDa and 100 kDa corresponding to a dimer (theoretical mass ~ 95 kDa). The broadband at the bottom of each lane corresponds to excess G-250, an anionic dye binding to the protein to ensure migration towards the cathode, added for non-denaturing gel analysis. This analysis was repeated independently once with similar results. Note the IDH1 R132S variant was not the subject of this study.* ***(e)/(f)*** *SEC-MALS analysis of IDH1 variants. The proteins were diluted to 1 mg/L in buffer containing 20 mM Tris, 100 mM NaCl (pH 7.4) and analysed using a Superdex 200 HR10/30 column. The combined results imply that the IDH1 variants are predominantly dimeric.* ***(g)*** *Magnified section of a non-denaturing MS spectrum (z = 20) of R132C (20 µM) showing it copurifies with two NADPH molecules. Note the presence of two adducts with 180 Da (*) and a 265 Da (**) mass shifts relative to the 2x NADPH complex. Conditions: ammonium citrate (200 mM, pH 7.5); cone-voltage: 100 V.* ***(h)*** *^{1}H NMR (700 MHz) studies showing copurification of NADPH with R132C. The protein was denatured by heating to 100°C.* ***(i)*** *Circular dichroism (CD) measurements in sodium phosphate buffer (20 mM, pH 8.0).* ***(j)*** *Summary table of T_ms as determined by DSF or CD.* ***(k)*** *Non-denaturing MS studies show that the higher mass adducts (arrows) are not removed by increasing the cone voltages suggesting that they are covalently linked.* ***(l)*** *LC/MS analysis of R132C before (blue) and after phosphatase treatment (black). After treatment, the signal at 47800 Da depletes and the signal at 47710 increases suggesting the observed covalent modification is due to (phospho)gluconoylation. Conditions: IDH1 R132C: 0.5 mg/mL, bovine alkaline phosphatase: 20 units; Tris (50 mM), $MgCl_2$ (5 mM), DTT (1 mM), glycerol (5%) at pH 7.5. Phosphatase treatment was conducted at 37°C for 1.5 h.*

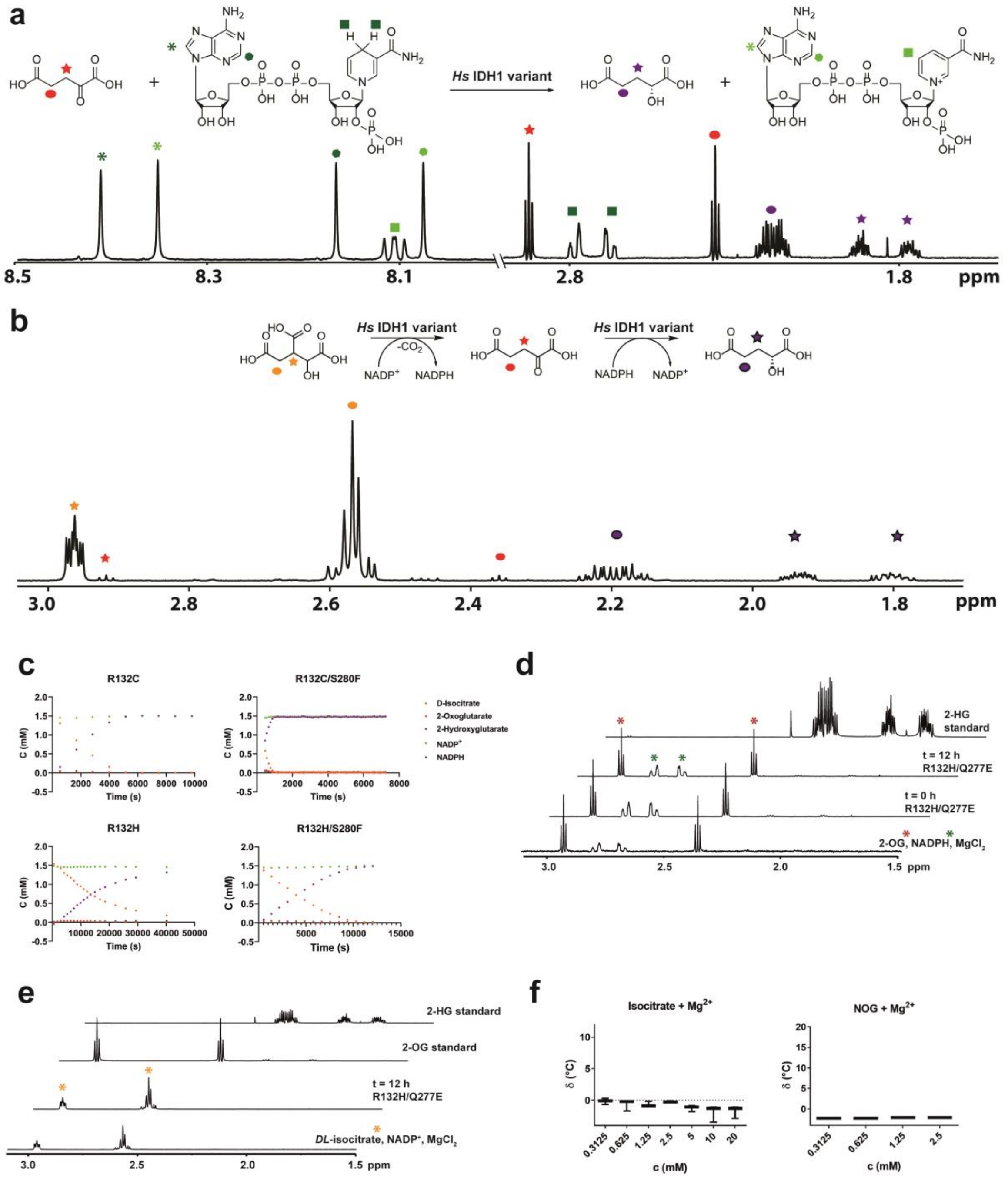

Supplementary Figure 2 | ^{1}H NMR analyses and T_m shift studies of IDH1 variants. (a) *IDH1 variant catalysed reduction of 2-OG to 2-HG and extract from a ^{1}H (700 MHz) spectrum showing corresponding signals. Conditions: 500 nM enzyme, 10 mM $MgCl_2$, 1.5 mM 2-OG, 1.5 mM NADPH, in 50 mM d_{11}-Tris buffer (90:10 H_2O:D_2O; pH 7.5).* ***(b)*** *IDH1 variant catalysed turnover of isocitrate to 2-OG then 2-HG, and extract of a ^{1}H spectrum showing the corresponding signals. Conditions: 750 nM enzyme, 10 mM $MgCl_2$, 3 mM DL-isocitrate, 1.5 mM $NADP^+$, in 50 mM d_{11}-Tris buffer (90:10 H_2O:D_2O; pH 7.5).* ***(c)*** *^{1}H NMR (700 MHz) analyses of the turnover of D-Isocitrate to 2-HG as catalysed by R132C, R132C/S280F, R132H, and R132H/S280F. Conditions: 750 nM protein, 10 mM $MgCl_2$, 1.5 mM D-ICT, and 1.5 mM $NADP^+$, in 50 mM d_{11}-Tris buffer (90:10 H_2O:D_2O; pH 7.5). Source data are provided as a Source Data file.* ***(d)-(e)*** *^{1}H NMR (700 MHz) time course studies show that R132H/Q277E is inactive/very poorly active.* ***(d)*** *^{1}H NMR (700 MHz) analyses of turnover of 2-OG as catalysed by R132H/Q277E. Conditions: 1 µM protein, 10 mM $MgCl_2$, 1.5 mM 2-OG, and 1.5 mM NADPH, in 50 mM d_{11}-Tris buffer (90:10 H_2O:D_2O; pH 7.5).* ***(e)*** *^{1}H NMR (700 MHz) analyses of the turnover of 2-OG as catalysed by R132H/Q277E. Conditions: 1 µM protein, 10 mM $MgCl_2$, 3 mM DL-isocitrate, and 1.5 mM $NADP^+$, in 50 mM d_{11}-Tris buffer (90:10 H_2O:D_2O; pH 7.5).* ***(f)*** *Influence of DL-isocitrate or NOG on the thermal stability of IDH1 R132H/Q277E variants by Differential Scanning Fluorimetry (DSF). Errors: standard error of the mean (n = 3 independent replicates measured on the same 96 well plate). Conditions: 3 µM enzyme, 10 mM $MgCl_2$, 50 mM Tris buffer, pH 7.5 (Dye: Sypro Orange). Source data are provided as a Source Data file.*

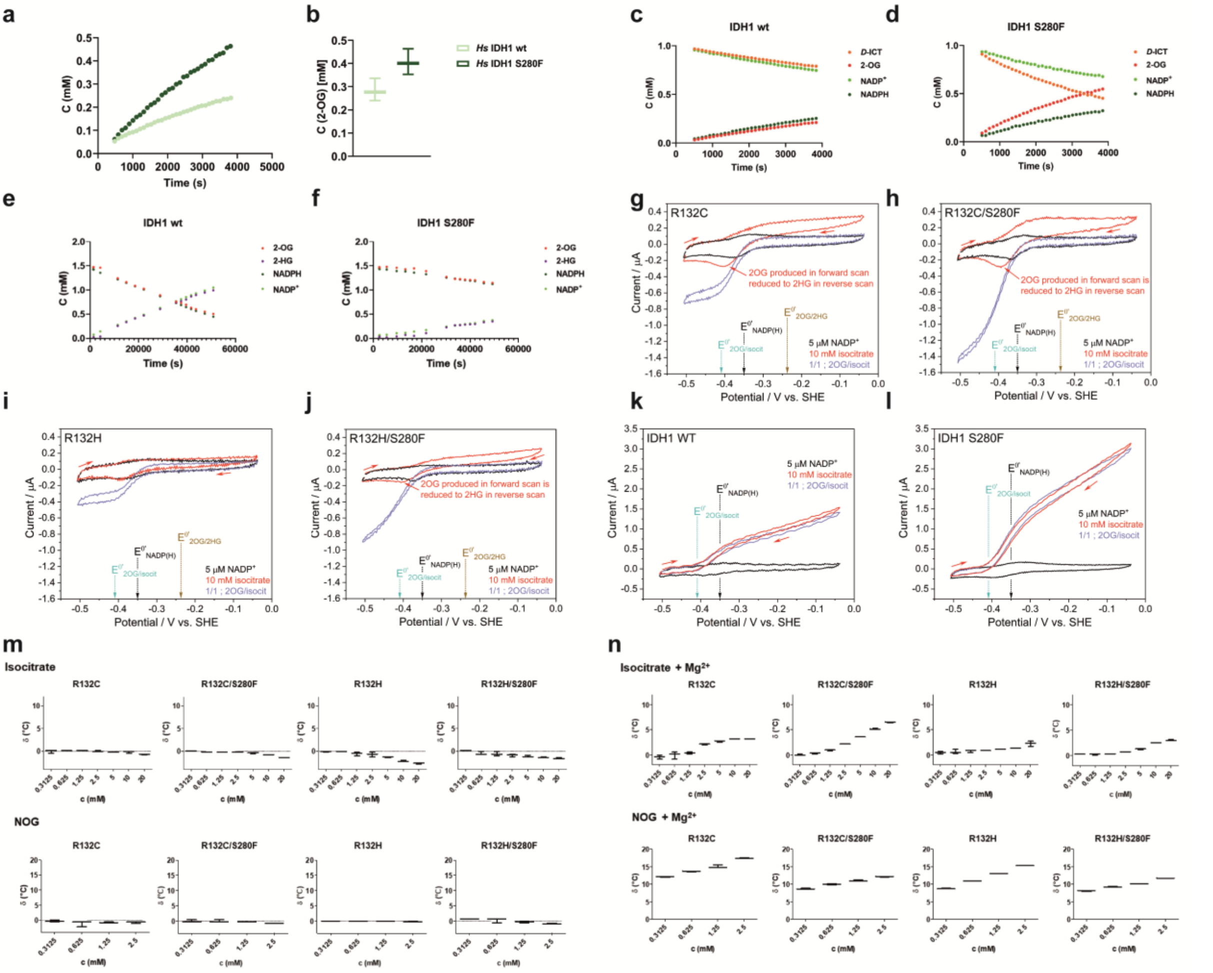

Supplementary Figure 3 | Studies of IDH1 variants by ¹H NMR time-course studies, cyclic voltammetry, and T_m shift analyses. (a)-(f) *¹H NMR (700 MHz) analyses of catalysis by IDH1 wt and the IDH1 S280F variant. Conditions: 10 mM $MgCl_2$, 50 mM d_{11}-Tris buffer (90:10 H_2O:D_2O; pH 7.5).* ***(a)*** *The S280F variant (dark green) differs in the rate of the conversion of DL-isocitrate to 2-OG compared to IDH1 wt (light green), reaching ~1.5-fold higher 2-OG levels after 1h* ***(b)****. Conditions: 10 nM enzyme, 500 µM DL-isocitrate, and 500 µM $NADP^+$. Errors: standard error of the mean (n = 3 independent replicates of ¹H time course experiments).* ***(c)/(d)*** *Turnover of D-isocitrate. Conditions: 10 nM enzyme, 500 nM D-ICT, and 500 nM $NADP^+$.* ***(e)/(f)*** *Turnover of 2-OG to 2-HG. Conditions: 1 µM enzyme, 1.5 mM 2-OG, and 1.5 mM NADPH; reaction was monitored over ~14 h.* ***(g)-(l)*** *Stationary cyclic voltammetry analyses to simultaneously measure the ability of wild-type IDH1 and five IDH1 variants to catalyse the oxidation of isocitrate and reduction of 2-oxoglutarate (2-OG)****.*** *Enzyme loading molar ratios: (****g–j****) FNR/neoIDH1; 1/2.5 (neoIDH1 represents any of the neomorphic IDH1 variants); (****k*** *and* ***l****) FNR/IDH1 wt or S280F; 8/1. Enzyme ratios were calculated based on IDH1 homodimer concentrations. $E^{0\prime}_{NADP(H)}$, $E^{0\prime}_{2\text{-}OG/isocit}$, and $E^{0\prime}_{2\text{-}OG/2\text{-}HG}$ denote formal potentials for the $NADP^+$/NADPH, 2-OG/DL-isocitrate, and 2-OG/2-HG couples, respectively.[3] Purple traces (1/1; 2-OG/DL-isocitrate) correspond to reactions with 10 mM 2-OG + 10 mM isocitrate. Conditions: stationary (FNR+E2)@ITO/PGE electrode (E2 represents a homodimeric IDH1 enzyme), electrode area 0.03 cm^2, scan rate 1 mV/s, temperature 25°C, volume 4 mL, pH 8 (20 mM each: MES, TAPS, CHES), 10 mM $MgCl_2$, 5 µM $NADP^+$.* ***(m)*** *T_m-shift analyses of IDH1 variants by DSF in the absence of magnesium ions. The influence of DL-isocitrate and NOG on the thermal stability of R132C, R132C/S280F, R132H, and R132H/S280F. Errors: standard errors of the mean (n = 3 independent replicates measured on the same 96 well plate). Conditions: 3 µM enzyme, in 50 mM tris buffer, pH 7.5 (Dye: Sypro Orange).* ***(n)*** *T_m-shift analyses of IDH1 variants by DSF in the presence of magnesium ions. The influence of DL-isocitrate and NOG on the thermal stability of R132C, R132C/S280F, R132H, and R132H/S280F. Errors: standard errors of the mean (n = 3 independent replicates measured on the same 96 well plate). Conditions: 3 µM enzyme, in 50 mM Tris buffer, pH 7.5 (Dye: Sypro Orange), 10 mM $MgCl_2$. Source data are provided as a Source Data file.*

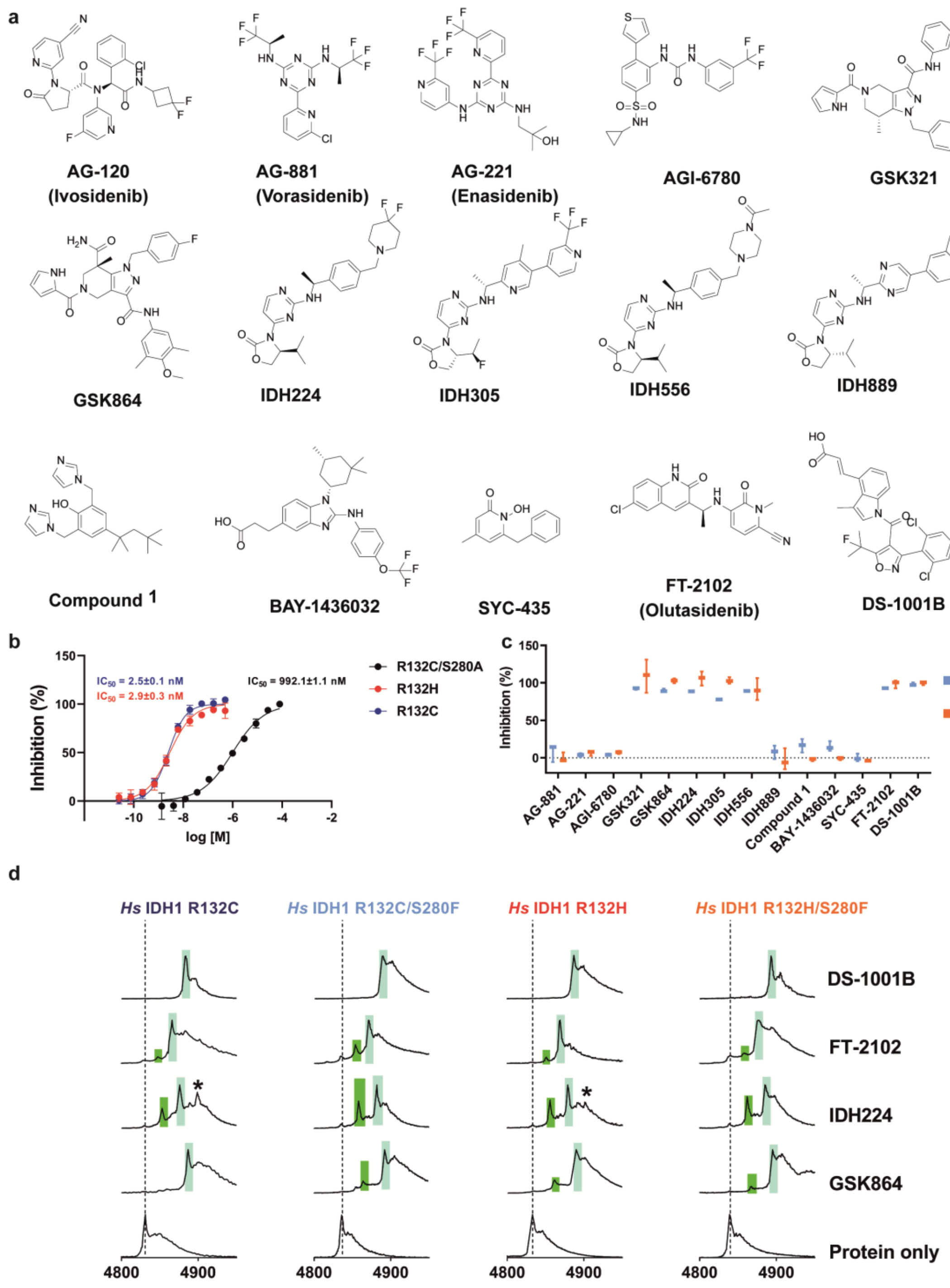

Supplementary Figure 4 | Inhibition and binding studies on IDH1 variants. (a) *Known IDH1 variant inhibitors[4,5,6,7,8,9,10,11] used in inhibition studies with R132C, R132C/S280F, R132C/S280A, R132H and R132H/S280F. Note that most of these inhibitors (likely) bind at the dimer-interface, with an exception being SYC-435.[12]* ***(b)*** *IC_{50} determination for ivosidenib with R132C/S280A. Errors: standard errors of the mean (n = 3 independent replicates measured on the same 96 well plate). Conditions: 100 mM Tris, 10 mM $MgCl_2$, 0.2 mM DTT, 0.005 $\%_{(v/v)}$ Tween 20, and 0.1 mg/mL bovine serum albumin (BSA), pH 8.0. Source data are provided as a Source Data file.* ***(c)*** *Inhibitor screening (%) with R132C/S280F and R132H/S280F (400 nM). Error bars: standard errors of the mean (n = 3 independent replicates measured on the same 96 well plate). Note that, like some, but not all of the allosterically binding inhibitors, SYC-435, which is reported to bind at the active site based on crystallographic studies[12], does not inhibit the S280F variants. Conditions: 100 mM Tris, 10 mM $MgCl_2$, 0.2 mM DTT, 0.005 $\%_{(v/v)}$ Tween 20, and 0.1 mg/mL bovine serum albumin (BSA), pH 8.0; 10 µM inhibitor was used. Source data are provided as a Source Data file.* ***(d)*** *Binding analyses with R132C, R132C/S280F, R132H, and R132H/S280F using non-denaturing mass spectrometry. Dashed line: IDH1 dimer (z = 20, with 2 NADPH molecules bound), bright green: one inhibitor bound, orange: two inhibitors bound. * indicates binding of a third inhibitor molecule, as observed at high inhibitor concentrations. Conditions: ammonium citrate (200 mM, pH 7.5). 20 µM IDH1 variant, 160 µM inhibitor, cone-voltage: 100 V.*

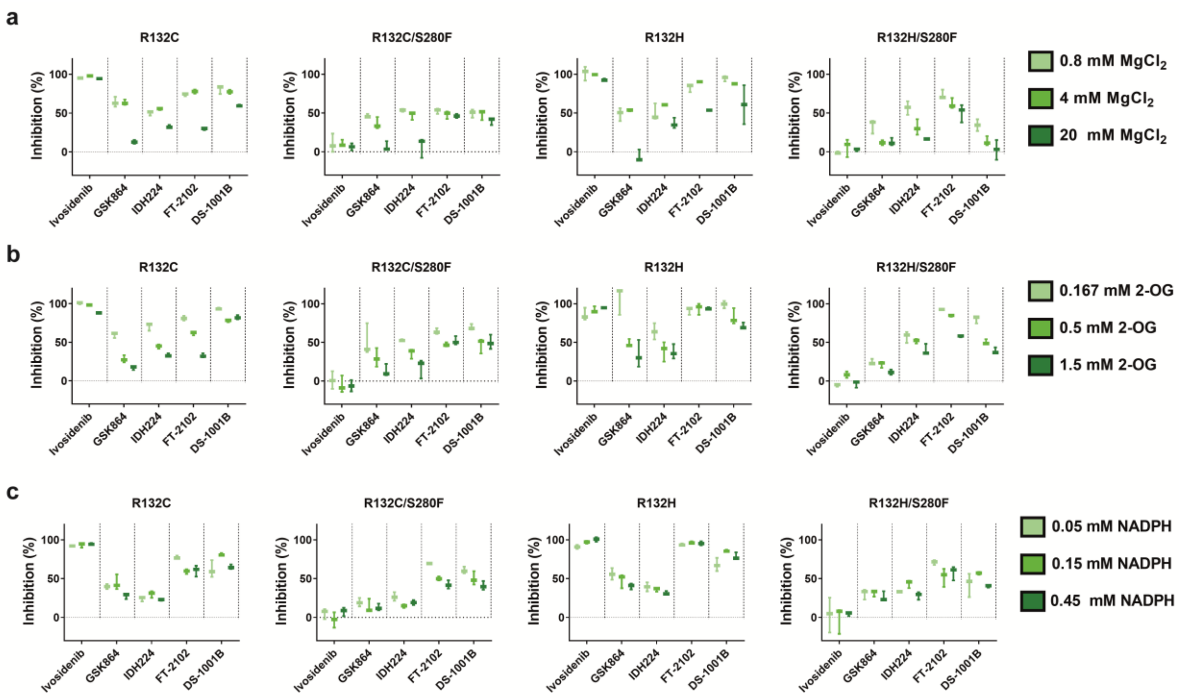

Supplementary Figure 5 | Competition studies on IDH1 variant inhibition. (a) *Inhibition of R132C, R132C/S280F, R132H, and R132H/S280F with varying $MgCl_2$ concentrations - the Mg^{2+} concentration influences inhibitor potency. Conditions: 400 nM enzyme, 200 nM inhibitor and varying concentrations of $MgCl_2$. 2-OG: 1.5 mM, NADPH: 0.3 mM.* ***(b)*** *Inhibition of R132C, R132C/S280F, R132H, and R132H/S280F with varying 2-OG concentrations - the 2-OG concentration influences inhibitor potency. Conditions: 400 nM enzyme, 200 nM inhibitor and 10 mM $MgCl_2$. NADPH 0.3 mM, 2-OG: varied.* ***(c)*** *Inhibition of R132C, R132C/S280F, R132H, and R132H/S280F with varying NADPH concentrations. Inhibitor potency is not substantially influenced by the NADPH concentration, except potentially for FT-2102. Conditions: 400 nM enzyme, 200 nM inhibitor and 10 mM $MgCl_2$. 2-OG: 1.5 mM, NADPH: varied. Buffer: 100 mM Tris, 0.2 mM DTT, 0.005 $\%_{(v/v)}$ Tween 20, and 0.1 mg/mL bovine serum albumin (BSA), pH 8.0. Error bars: standard errors of the mean (n = 3 independent replicates measured on the same 96 well plate). Source data are provided as a Source Data file.*

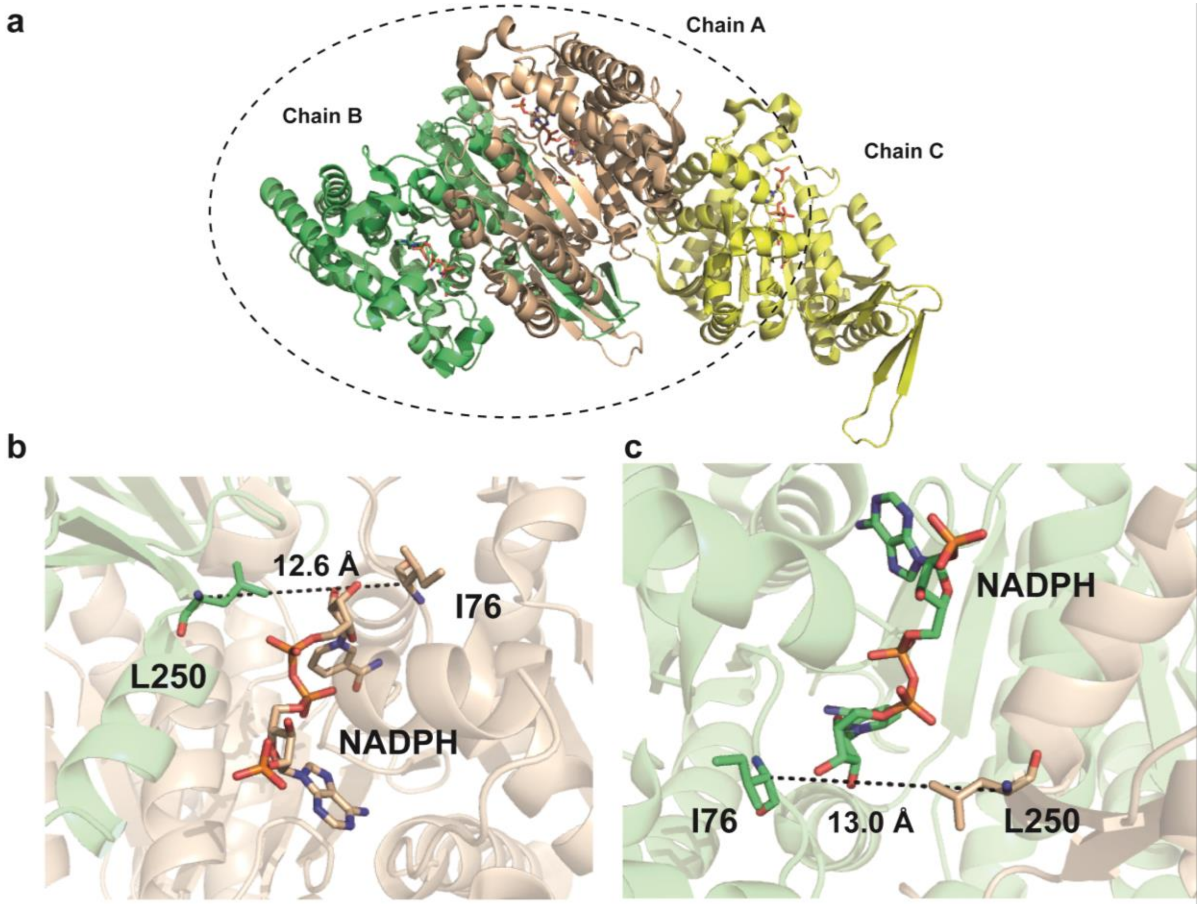

Supplementary Figure 6 | Crystal structure analysis of R132C/S280F complexed with NADPH, 2-OG, and calcium. (a) *Ribbon view of the asymmetric unit from a crystal structure of R132C/S280F (PDB: 7PJM, 2.1 Å resolution) complexed with NADPH, 2-OG, and calcium ions, likely in a closed active conformation. There are 3 protein molecules in the asymmetric unit (chain A (wheat), chain B (green), chain C (yellow)). Non-denaturing gel and SEC-MALS analyses imply that R132C/S280F is predominantly dimeric in solution. Chains A and B form an apparent dimer in the asymmetric unit; chain C interacts with chain C from another asymmetric unit forming an apparent dimer. Each of the two active sites in the dimer is composed of residues from both chains A and B. The distance between the I76 and L250 Cα carbons, which is used to measure the width of the active site cleft[13], is 12.6 Å (**b**; I76 (chain A), L250 (chain B)) or 13.0 Å (**c**; I76 (chain B), L250 (chain A)).*

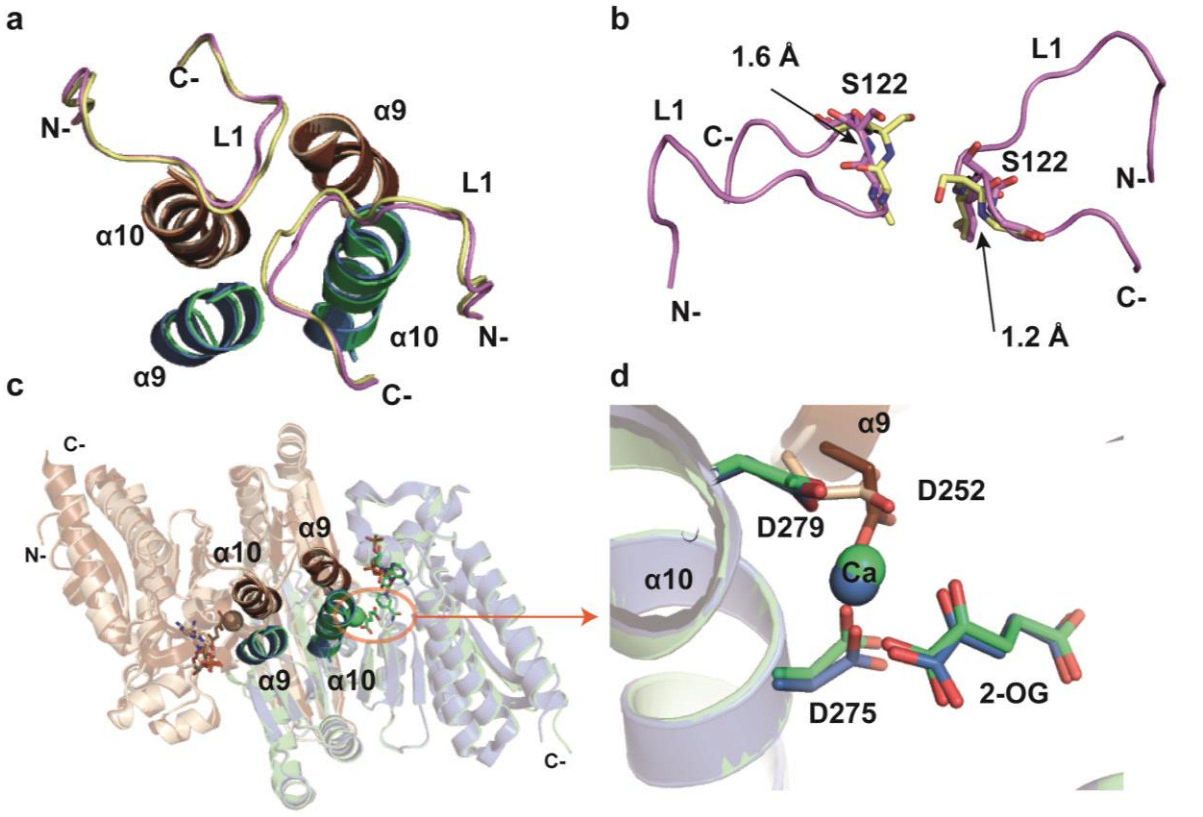

Supplementary Figure 7 | Views from crystallographic analyses of R132C/S280F complexed with NADPH, 2-OG, and calcium, likely in its closed active conformation (a-d; PDB: 7PJM). (a) *Ribbon view of the dimer-interface and L1 loop of R132C/S280F (2.1 Å resolution; chain A: wheat, chain B: green)) superimposed with a view from a structure of R132H (PDB: 4KZO[1]; chain A: brown, chain B: blue).* ***(b)*** *Close-up view showing the movement of the L1 loop, which partially covers the inhibitor binding pocket (violet: R132C/S280F, yellow: R132H) and the conformational change associated with residue S122 relative to R132H. The distances are measured between the Cα atoms of S122 of R132C/S280F and S122 of R132H.* ***(c-d)*** *Views from a crystal structure of R132C/S280F highlight the metal-binding site.* ***(c)*** *Ribbon view: a crystal structure of R132C/S280F (PDB: 7PJM, 2.1 Å resolution; chain A: wheat, chain B: green) superimposed with a view from a crystal structure of IDH1 R132H (PDB: 4KZO[1]; chain A: brown, chain B: blue; main chain RMSD: 0.486 Å). Orange circle: metal-binding site. L1 loop: violet.*

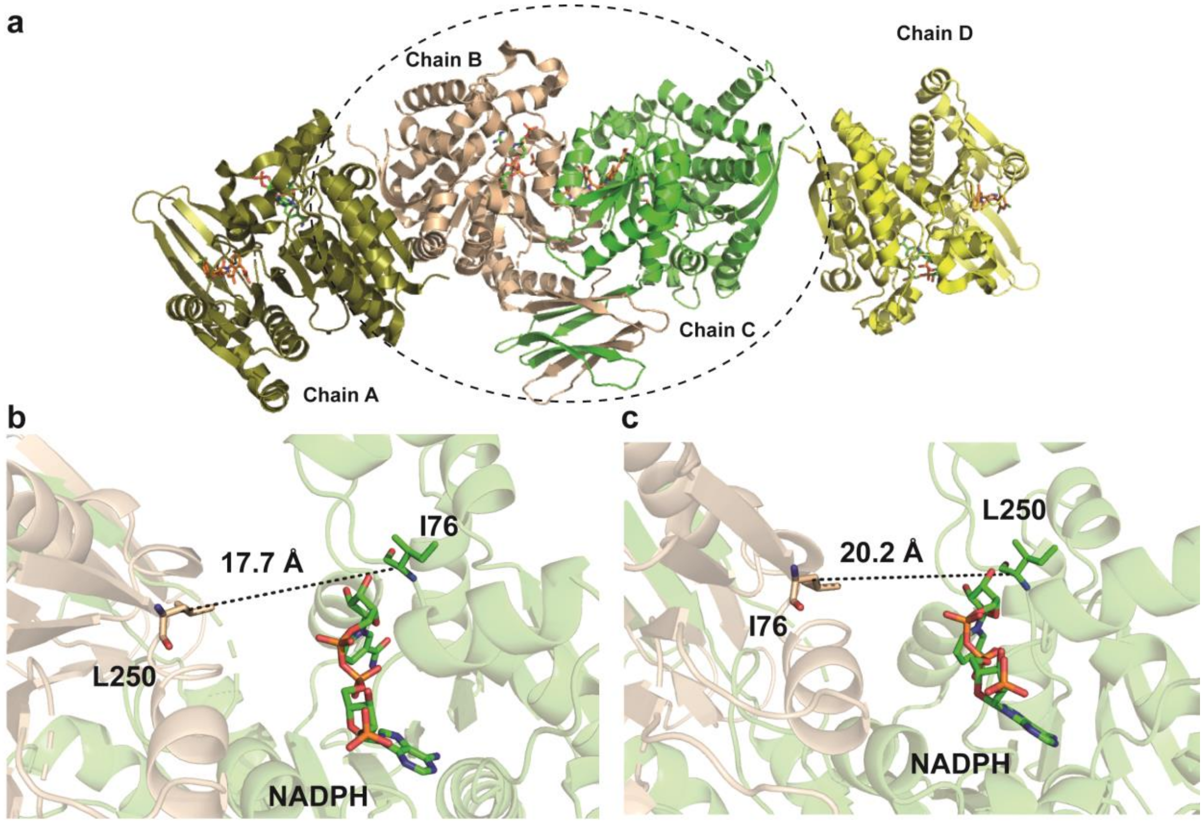

Supplementary Figure 8 | Crystal structure analysis of R132C/S280F complexed with NADPH and DS-1001B. (a) *Ribbon view from a crystal structure of IDH1 R132C/S280F (PDB: 7PJN, 2.45 Å resolution) complexed with 4 x NADPH and 4 x DS-1001B (orange) likely in an open inactive conformation. There are four monomers in the asymmetric unit (chain A (olive), chain B (wheat), chain C (green), chain D (yellow)). Non-denaturing gel analyses and SEC-MALS analyses imply that R132C/S280F is predominantly dimeric in solution. Chain B and chain C form an apparent dimer in the asymmetric unit, while chain A forms an apparent dimer with chain D from another asymmetric unit. Each of the two active sites in the dimer is composed of residues from both chains A and B. The distance between the I76 and L250 Cα carbons which is used to measure the width of the active site cleft is 17.7 Å (**b;** L250, chain B and I76, chain C) or 20.2 Å (**c;** I76, chain B and L250, chain C).*

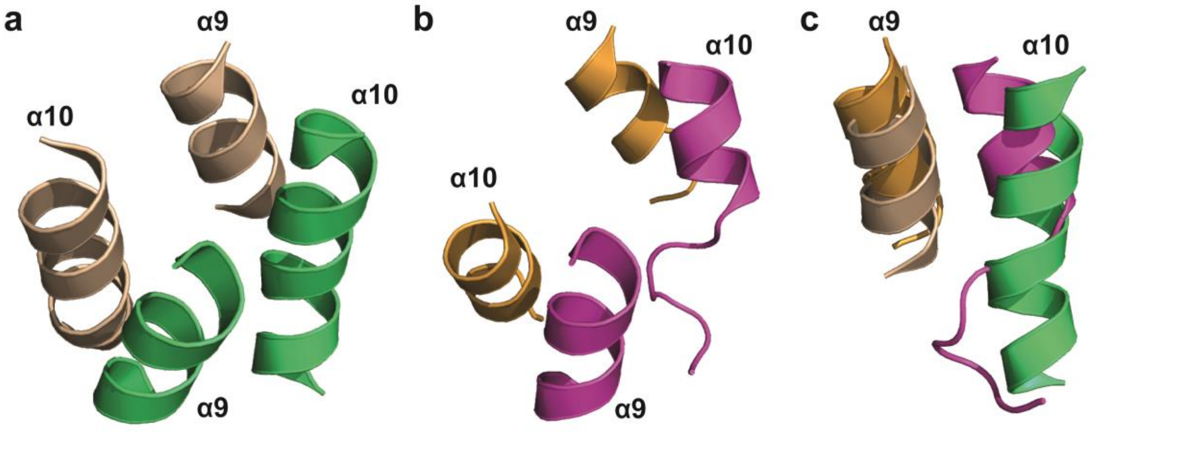

Supplementary Figure 9 | Views from the dimer-interface of R132C/S280F. (a) *Ribbon view from α9 and α10 of R132C/S280F (2.1 Å resolution; chain A: wheat, chain B: green; PDB: 7PJM)) in the closed (likely active) conformation.* ***(b)*** *Ribbon view from α9 and α10 of R132C/S280F (2.45 Å resolution; chain A: orange, chain B: purple; PDB: 7PJN)) in the open (likely inactive) conformation.* ***(c)*** *Ribbon view from α9 and α10 of R132C/S280F in the closed (active) conformation superimposed with α9 and α10 in the open likely (inactive) conformation (main chain RMSD: 4.169 Å).*

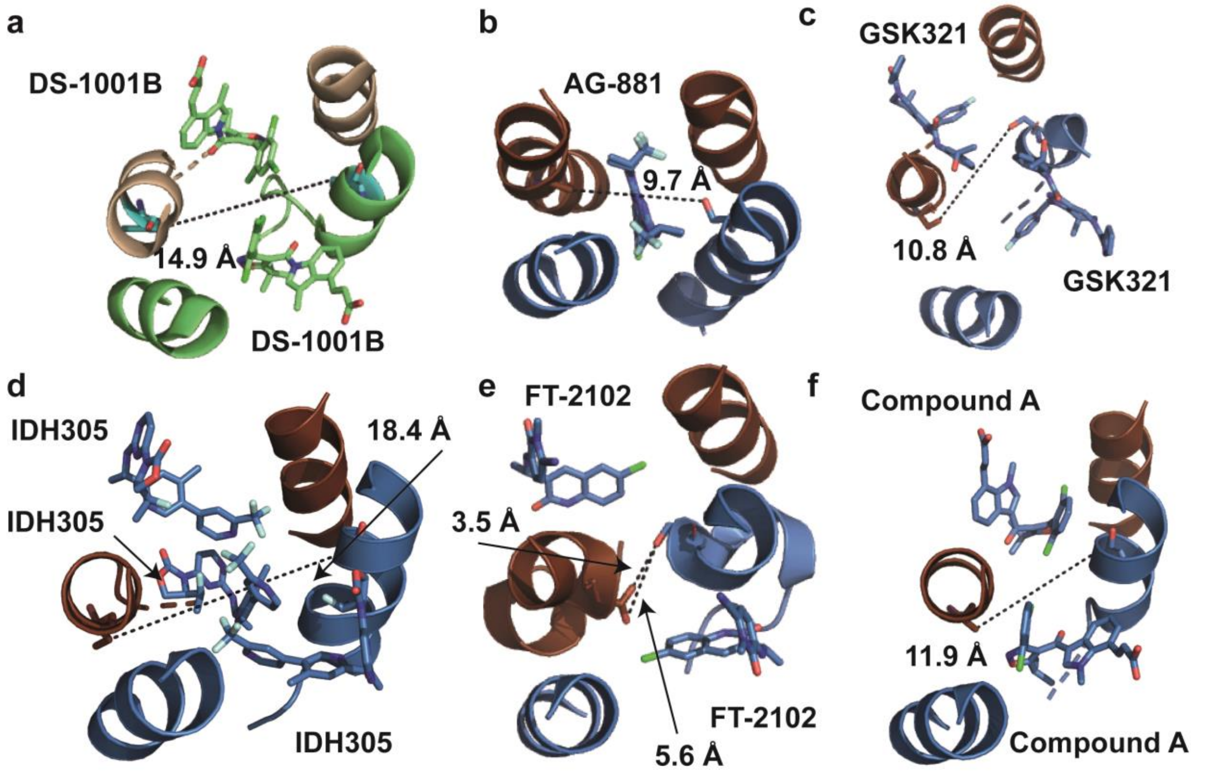

Supplementary Figure 10 | Views from crystal structures of IDH1 variants complexed with inhibitors. (a-f) *Ribbon views from crystal structures of IDH1 variants complexed with DS-1001B* ***(a)****, AG-881* ***(b)****, GSK321* ***(c)****, IDH305* ***(d)****, FT-2102* ***(e)****, Compound A* ***(f)*** *measuring the distance between residue 280 in the 2 monomers (closest C atoms). (****a)*** *One monomer of the assigned dimer is in wheat, the other monomer in green. (****b)-(f).*** *One monomer is in brown, the other monomer in blue.* ***(a)*** *Ribbon view from a crystal structure of R132C/S280F complexed with DS-1001B (7PJN) manifests a distance of 14.9 Å between F280 in the 2 monomers.* ***(b)*** *Ribbon view from a crystal structure of R132H complexed with AG-881 (6ADG[14]), which manifests a distance of 9.7 Å between S280 in the 2 monomers.* ***(c)*** *Ribbon view from a crystal structure of R132H complexed with GSK321 (5DE1[6]) which manifests a distance of 10.8 Å between S280 in the 2 monomers.* ***(d)*** *Ribbon view from a crystal structure of R132H complexed with IDH305 (6B0Z[7]) which manifests a distance of 18.4 Å between S280 in the 2 monomers.* ***(e)*** *Ribbon view from a crystal structure of R132H complexed with FT-2102 (6U4J[10]) which manifests a distance of 3.5 Å or 5.6 Å (other conformer) between S280 in the 2 monomers.* ***(f)*** *Ribbon view from a crystal structure of R132C complexed with Compound A, a DS-1001B analogue, (6IO0[11]) which manifests a distance of 11.9 Å between S280 in the 2 monomers.*

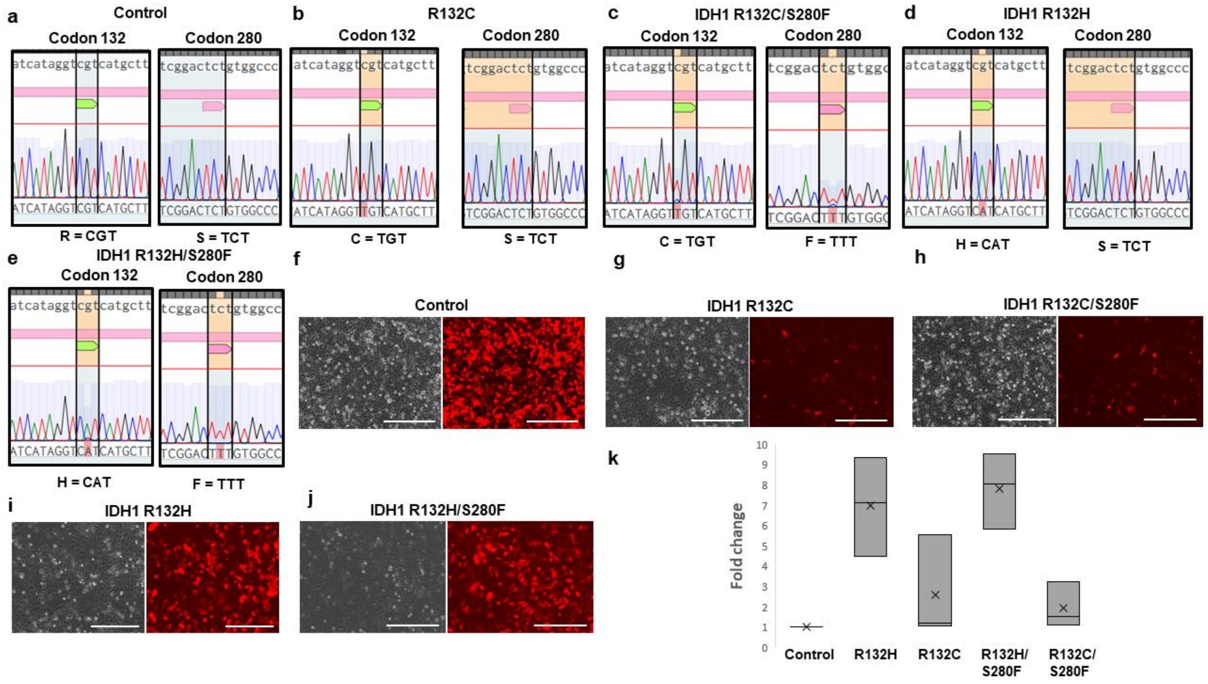

Supplementary Figure 11 | Lentiviral transduction of LN18 cells with IDH1 variants. (a)-(e) *Detection of either R132H or R132C, the double R132H/S280F, or the R132C/S280F mutations of IDH1 in lentiviral transduced cells. Sequencing electropherograms show cDNA of IDH1 in a region around codon 132 or codon 280. Arrows indicate nucleotides altered in IDH1 R132H (c. 395 CGT > CAT), in IDH1 R132C (c.394 CGT>TGT) and in IDH1 S280F (c.839 TCT>TTT) mutations.* ***(f)-(j)*** *TdTomato expression in LN18 cells transduced with-mutant IDH1 bicistronic lentiviral vectors. LN18 cells were transduced with bicistronic lentiviral vectors, leading to the expression of mutant IDH1 and TdTomato; Td tomato expression was used as a control of transduction efficiency. (**f**) Cells expressing only TdTomato (control); cells expressing either TdTomato and IDH1 R132C (**g**), R132C/S280F (**h**), R132H (**i**), or R132H/S280F (**j**) transgenes. λ (TdTomato excitation) = 554 nm. Similar tdTomato expression was observed at least 3 times.* ***(k)*** *TaqMan plot showing levels of expression of mutant IDH1. Overexpression of each transgene was quantified using qRT-PCR. It was statistically analysed using a one-way ANOVA test (n = 3 biological replicates). Errors: standard deviation of the mean. R132H (p-value: 0.0089), R132C (p-value: 0.6778), R132H/S280F (p-value: 0.0038) and R132C/S280F (p-value: 0.919). Source data are provided as a Source Data file.*

Supplementary Tables

a

**Enzyme**	**IDH1 wt**		
	**K_M (µM)**	**k_{cat} (s^{-1})**	**k_{cat}/K_M ($s^{-1}.\mu M^{-1}$)**
***DL*-Isocitrate**	31±3	43±1	1.4±0.2
**$NADP^+$**	10.1±0.7	43.2±0.7	4.3±0.4
**$MgCl_2$**	18±2	37±1	2.1±0.3
	**IDH1 S280F**		
	**K_M (µM)**	**k_{cat} (s^{-1})**	**k_{cat}/K_M ($s^{-1}.\mu M^{-1}$)**
***DL*-Isocitrate**	12±1	33.4±0.9	2.8±0.3
**$NADP^+$**	7.4±0.7	31.3±0.6	4.2±0.5
**$MgCl_2$**	24±3	33±1	1.4±0.2

b

**Enzyme**	**IDH1 wt**		
	**K_M (µM)**	**k_{cat} (s^{-1})**	**k_{cat}/K_M ($s^{-1}.\mu M^{-1}$)**
**2-oxoglutarate**	792±103	$(4.4\pm0.1)*10^{-2}$	$(5.6\pm0.8)*10^{-5}$
**NADPH**	7±2	$(3.0\pm0.1)*10^{-2}$	$(4\pm1)*10^{-3}$
**$MgCl_2$**	$(1.4\pm0.1)*10^{3}$	$(4.1\pm0.1)*10^{-2}$	$(2.9\pm0.3)*10^{-5}$
	**IDH1 S280F**		
	**K_M (µM)**	**k_{cat} (s^{-1})**	**k_{cat}/K_M ($s^{-1}.\mu M^{-1}$)**
**2-oxoglutarate**	123±4	$(5.1\pm0.5)*10^{-3}$	$(4.1\pm0.5)*10^{-5}$
**NADPH**	3.0±0.3	$(4.9\pm0.2)*10^{-3}$	$(1.6\pm0.2)*10^{-3}$
**$MgCl_2$**	15±4	$(4.2\pm0.2)*10^{-3}$	$(2.8\pm0.9)*10^{-4}$

Supplementary Table 1 | Comparison of Michaelis-Menten parameters for IDH1 wt and IDH1 S280F using a spectrophotometric NADPH absorption assay. *Conditions: 100 mM Tris, 10 mM $MgCl_2$, 0.2 mM DTT, 0.005 %(v/v) Tween 20, and 0.1 mg/mL bovine serum albumin (BSA), pH 8.0. Enzyme concentrations: 1 nM (**a**) or 2.5 µM (**b**). (a) Michaelis Menten parameters for the oxidative decarboxylation of isocitrate. Errors: standard error of the mean (n = 3). (b) Michaelis Menten parameters for the reduction of 2-OG to 2-HG. Errors: standard error of the mean (n = 3).*

Site-Directed Mutagenesis	
**Primer**	**Sequence**
R132H/S280F_forward	GGTGACGTGCAGTCGGACTTTGTGGCCCAA
R132H/S280F_reverse	TTGGGCCACAAAGTCCGACTGCACGTCACC
R132C_forward	CATAGGTTGTCATGCTTATGGGGATC
R132C_reverse	GCATGACAACCTATGATGATAGGTTTTACCC
R132C/S280F_forward	GGTGACGTGCAGTCGGACTTTGTGGCCCAA
R132C/S280F_reverse	TTGGGCCACAAAGTCCGACTGCACGTCACC
R132C/S280A_forward	GTCGGACGCTGTGGCCCAAGCTATG
R132C/S280A_reverse	GCCACAGCGTCCGACTGCACGTC
**Lentiviral Work**	
**Primer**	**Sequence**
Primer1_forward	GGTGACGTGCAGTCGGACTTTGTGGCCCAA
Primer1_reverse	TCTTTCCCCTGCACTGTACC
Primer2_forward	TATACCCGGGCCTATGTCCAAAAAAATCAGTGGCGG
Primer2_reverse	TCCACATAGCGTAAAAGGAGCA
Primer3_forward	GACCAAGTCACCAAGGATGC
Primer3_reverse	GCTTTGCTCTGTGGGCTAA

Supplementary Table 2 | Primer sequences used for site-directed mutagenesis and lentiviral work.

Protein	*Mw (theoretical)*	*Mw (observed)*	*Difference in Mw*
IDH1 R132H	*47705.37*	*47574.4*	*-130.97*
IDH1 R132H/S280F	*47765.47*	*47632.5*	*-132.97*
IDH1 R132C	*47671.37*	*47539.4*	*-131.97*
IDH1 R132C/S280F	*47731.47*	*47599.7*	*-131.77*
IDH1 R132C/S280A	*47655.37*	*47524.2*	*-131.17*
IDH1 wt	*47724.42*	*47591.8*	*-132.62*
IDH1 S280F	*47784.52*	*47651.31*	*-133.21*
IDH1 R132H/Q277E	*47706.36*	*47575.5*	*-130.86*

Supplementary Table 3 | Analyses of IDH1 variants by LC/MS*. The loss of a ~130 Da fragment corresponds to N-terminal methionine loss.*

Datasets	*IDH1:NADPH:CA:AKG (PDB ID: 7PJM)*	*IDH1:NADPH:DS1001B (PDB ID: 7PJN)*
Data Collection (T in K)	*cryo-MX (100)*	*cryo-MX (100)*
Beamline (Wavelength, Å)	*DLS I24 (0.96863)*	*DLS I03 (0.97933)*
Detector	*DECTRIS PILATUS 6M*	*DECTRIS EIGER2 XE 16M*
Data Processing	*Xia2*	*Xia2*
Space group	*C 2 2 2_1*	*P 2_1 2_1 2_1*
Cell dimensions		
a,b,c (Å)	*96.58, 273.01, 117.42*	*80.18, 153.05, 164.01*
α, β, γ (º)	*90, 90, 90*	*90, 90, 90*
No. of molecules/ASU	*3*	*4*
No. reflections	*90634 (6591)*	*74987 (5470)**
Resolution (Å)	*71.950 - 2.100 (2.150 - 2.100)**	*55.950- 2.450 (2.510 - 2.450)**
R_{meas}(I)	*0.110 (2.469)**	*0.096 (2.297)**
I/σI	*13.400 (1.1)**	*18.900 (1.2)**
CC-half	*0.999 (0.550)**	*1.000 (0.513)*
Completeness (%)	*99.96 (99.87)**	*99.91 (99.61)**
Multiplicity	*13.2 (12.7)**	*13.7 (13.3)**
Wilson B value (Å^2)	*47.61*	*65.35*
Refinement	*PHENIX*	*PHENIX*
R_{work}/R_{free+}	*0.1805/0.2098*	*0.1861 /0.2259*
No. atoms	*10275*	*12975*
- *Enzyme*	*9730*	*12432*
- *Ligand*	*204*	*433*
- *Water*	*341*	*110*
Average B-factors	*59.91*	*74.79*
- *Enzyme (Å^2)*	*59.99*	*74.68*
- *Ligand (Å^2)*	*64.88*	*80.48*
- *Water (Å^2)*	*54.66*	*64.20*
R.m.s deviations		
- *Bond lengths (Å)*	*0.003*	*0.004*
- *Bond angles (º)*	*0.52*	*0.68*

**Highest resolution shell in parentheses. DLS = Diamond Light Source.*

Supplementary Table 4 | Data collection and refinement statistics for structures PDBID 7PJM and PDBID 7PJN.

2-OG	p-value
K_M (R132C) vs K_M (R132C/S280F)	0.0119
K_M (R132H) vs K_M (R132H/S280F)	0.0005
k_{cat}/K_M (R132C) vs k_{cat}/K_M (R132C/S280F)	0.0147
k_{cat}/K_M (R132H) vs k_{cat}/K_M (R132H/S280F)	0.0023
K_M (IDH1 wt) vs K_M (S280F)	0.0029
**$MgCl_2$**	
K_M (R132C) vs K_M (R132C/S280F)	0.0497
K_M (R132H) vs K_M (R132H/S280F)	0.0283
k_{cat}/K_M (R132C) vs k_{cat}/K_M (R132C/S280F)	0.0137
k_{cat}/K_M (R132H) vs k_{cat}/K_M (R132H/S280F)	0.0064
K_M (IDH1 wt) vs K_M (S280F) (2-OG reduction)	0.0003
**Isocitrate**	
K_M (IDH1 wt) vs K_M (S280F)	0.0039
k_{cat}/K_M (IDH1 wt) vs k_{cat}/K_M (280F)	0.0178

Supplementary Table 5 | Significance testing of comparisons of kinetic parameters using unpaired, two-sided t-tests.

Supplementary References

1. Rendina, A. R. *et al.* Mutant IDH1 enhances the production of 2-hydroxyglutarate due to its kinetic mechanism. *Biochemistry* **52**, 4563–4577 (2013).
2. Yen, K. *et al.* AG-221, a First-in-Class Therapy Targeting Acute Myeloid Leukemia Harboring Oncogenic IDH2 Mutations. *Cancer Discov* **7**, 478–493 (2017).
3. Herold, R. A. *et al.* Exploiting Electrode Nanoconfinement to Investigate the Catalytic Properties of Isocitrate Dehydrogenase (IDH1) and a Cancer-Associated Variant. *J. Phys. Chem. Lett.* **12**, 6095–6101 (2021).
4. Urban, D. J. *et al.* Assessing inhibitors of mutant isocitrate dehydrogenase using a suite of pre-clinical discovery assays. *Sci Rep* **7**, 12758 (2017).
5. Ma, R. & Yun, C.-H. Crystal structures of pan-IDH inhibitor AG-881 in complex with mutant human IDH1 and IDH2. *Biochemical and Biophysical Research Communications* **503**, 2912–2917 (2018).
6. Okoye-Okafor, U. C. *et al.* New IDH1 mutant inhibitors for treatment of acute myeloid leukemia. *Nat Chem Biol* **11**, 878–886 (2015).
7. Cho, Y. S. *et al.* Discovery and Evaluation of Clinical Candidate IDH305, a Brain Penetrant Mutant IDH1 Inhibitor. *ACS Med Chem Lett* **8**, 1116–1121 (2017).
8. Pusch, S. *et al.* Pan-mutant IDH1 inhibitor BAY 1436032 for effective treatment of IDH1 mutant astrocytoma in vivo. *Acta Neuropathol* **133**, 629–644 (2017).
9. Liu, Z. *et al.* Inhibition of Cancer-Associated Mutant Isocitrate Dehydrogenases: Synthesis, Structure–Activity Relationship, and Selective Antitumor Activity. *J. Med. Chem.* **57**, 8307–8318 (2014).
10. Caravella, J. A. *et al.* Structure-Based Design and Identification of FT-2102 (Olutasidenib), a Potent Mutant-Selective IDH1 Inhibitor. *J Med Chem* **63**, 1612–1623 (2020).

11. Machida, Y. *et al.* A Potent Blood-Brain Barrier-Permeable Mutant IDH1 Inhibitor Suppresses the Growth of Glioblastoma with IDH1 Mutation in a Patient-Derived Orthotopic Xenograft Model. *Mol Cancer Ther* **19**, 375–383 (2020).
12. Zheng, B. *et al.* Crystallographic Investigation and Selective Inhibition of Mutant Isocitrate Dehydrogenase. *ACS Med. Chem. Lett.* **4**, 542–546 (2013).
13. Xu, X. *et al.* Structures of Human Cytosolic NADP-dependent Isocitrate Dehydrogenase Reveal a Novel Self-regulatory Mechanism of Activity. *J. Biol. Chem.* **279**, 33946–33957 (2004).
14. Ma, R. & Yun, C.-H. Crystal structures of pan-IDH inhibitor AG-881 in complex with mutant human IDH1 and IDH2. *Biochem Biophys Res Commun* **503**, 2912–2917 (2018).

Uncropped gel, Figure S1, a

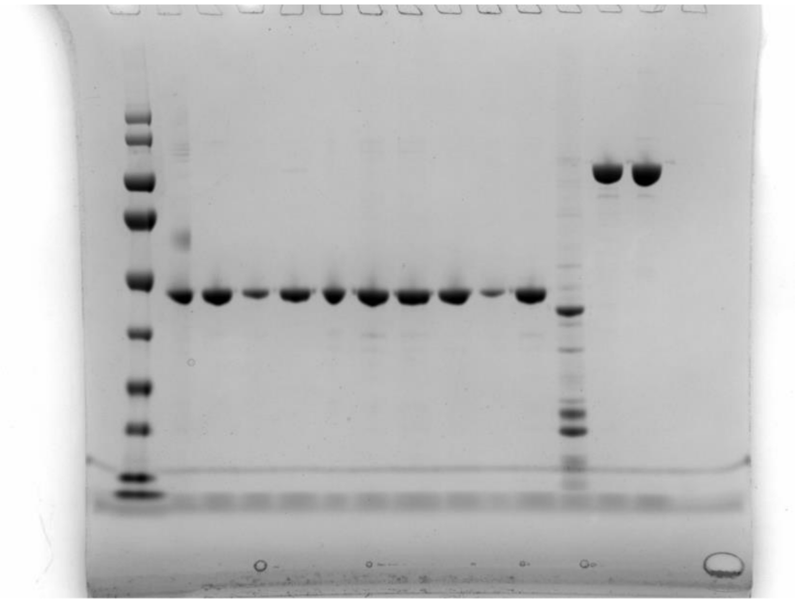

Uncropped gel, Figure S1, d

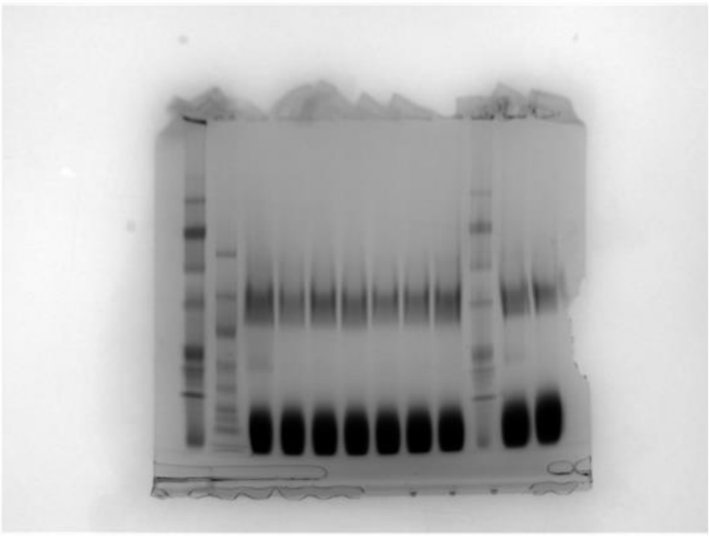

Supplement: Supplementary file 1 — Supplementary Info File #1 [file 41467_2022_32436_MOESM1_ESM.pdf]
